# Development of multivariable models to predict perinatal depression before and after delivery using patient reported survey responses at weeks 4–10 of pregnancy

**DOI:** 10.1186/s12884-022-04741-9

**Published:** 2022-05-26

**Authors:** Jenna M. Reps, Marsha Wilcox, Beth Ann McGee, Marie Leonte, Lauren LaCross, Kevin Wildenhaus

**Affiliations:** 1 Janssen Research & Development, Titusville, NJ USA; 2 BabyCenter, San Francisco, CA USA

**Keywords:** Patient-level prediction, Perinatal depression, Machine learning, Model development

## Abstract

**Background:**

Perinatal depression is estimated to affect ~ 12% of pregnancies and is linked to numerous negative outcomes. There is currently no model to predict perinatal depression at multiple time-points during and after pregnancy using variables ascertained early into pregnancy.

**Methods:**

A prospective cohort design where 858 participants filled in a baseline self-reported survey at week 4–10 of pregnancy (that included social economics, health history, various psychiatric measures), with follow-up until 3 months after delivery. Our primary outcome was an Edinburgh Postnatal Depression Score (EPDS) score of 12 or more (a proxy for perinatal depression) assessed during each trimester and again at two time periods after delivery. Five gradient boosting machines were trained to predict the risk of having EPDS score >  = 12 at each of the five follow-up periods. The predictors consisted of 21 variables from 3 validated psychometric scales. As a sensitivity analysis, we also investigated different predictor sets that contained: i) 17 of the 21 variables predictors by only including two of the psychometric scales and ii) including 143 additional social economics and health history predictors, resulting in 164 predictors.

**Results:**

We developed five prognostic models: PND-T1 (trimester 1), PND-T2 (trimester 2), PND-T3 (trimester 3), PND-A1 (after delivery 1) and PND-A2 (delayed onset after delivery) that calculate personalised risks while only requiring that women be asked 21 questions from 3 validated psychometric scales at weeks 4–10 of pregnancy. C-statistics (also known as AUC) ranged between 0.69 (95% CI 0.65–0.73) and 0.77 (95% CI 0.74–0.80). At 50% sensitivity the positive predictive value ranged between 30%-50% across the models, generally identifying groups of patients with double the average risk. Models trained using the 17 predictors and 164 predictors did not improve model performance compared to the models trained using 21 predictors.

**Conclusions:**

The five models can predict risk of perinatal depression within each trimester and in two post-natal periods using survey responses as early as week 4 of pregnancy with modest performance. The models need to be externally validated and prospectively tested to ensure generalizability to any pregnant patient.

**Supplementary Information:**

The online version contains supplementary material available at 10.1186/s12884-022-04741-9.

## Background

Perinatal depression (PND) is depression that occurs during or shortly after pregnancy and research has shown it to be a cause of numerous negative outcomes for the affected women’s children [1–3]. It is estimated to impact approximately 12% of pregnant women [4] and there is a lack of methodology to predict those at risk in the general population [1]. If predictions were available that could identify the higher risk patient population for different time-points during and shortly after pregnancy, then potential interventions could be developed to reduce this number through prevention and early interception of PND and improve health and wellbeing outcomes for both pregnant women and their children. For example, if certain pregnant women were identified at the beginning of pregnancy as being high risk of developing depression during trimester 3, then the healthcare provider could plan a future meeting to screen for depression during trimester 3. This could lead to earlier diagnosis and treatment of PND.

Researchers have identified numerous risk factors of perinatal or postpartum depression, including various psychological factors such as mental health issues prior to pregnancy [5], state and trait anxiety [6], poor relationships, stressful events and negative attitudes towards pregnancy [7]. The type of delivery such as emergency caesarean has also been linked to postpartum depression [8] as well as social support [7] and being a housewife [9]. Although many predictors have been identified, there is a lack of clinically useful predictive models that can be applied during the early stage of pregnancy to identify women at high risk for PND.

Examples of published models that predict postpartum depression include a logistic regression using antenatal variables such as age, marital status, occupational status, history of psychiatric disease, perceived social isolation and psychological distress during pregnancy, which was able to predict postpartum depression 4 month postpartum with a positive predictive value of 30%, sensitivity of 79% and specificity of 50% [10]. Another study developed a logistic regression model to predict postpartum depression 6–8 weeks postpartum using 17 variables self-reported in the third trimester and obtained a sensitivity of 33%, specificity of 87% and positive predictive value of 35% [11]. The Brisbane Postnatal Depression Index included antenatal and postnatal variables to predict postpartum depression (16 weeks postpartum) and obtained slightly better performance with a 36.3% sensitivity, 92% specificity and a 40% positive predictive value [12]. These models generally require variables collected during the mid to later stages of pregnancy, limiting their application to later stages of the pregnancy. In addition, they tend to focus on predicting depression after delivery. However, a recent study identified 80% onset in pregnancy, further highlighting the importance of early identification of risk [13].

In this paper we aim to develop clinically useful models that can predict depression during each trimester (weeks 12/13, 21 and 32) and at weeks 4 and 12 post-delivery using variables that are ascertained in weeks 4–10 of pregnancy. The clinical utility of the models is to be able to provide women who have just found out they are pregnant with a survey that can be answered and used to identify whether they are high risk of developing future depression during each trimester of pregnancy, shortly after delivery and 12 weeks after delivery. Pregnant women identified as being high-risk of developing depression during future time periods can then be scheduled for future depression screening during the time-period they are identified as high risk to ensure the depression is diagnosed early and treatment (e.g., SSRIs suitable during pregnancy) is provided to those who need it.

## Materials & methods

### Prediction questions

The prediction question answered in this paper is:

Within pregnant women, predict a self-reported Edinburgh Postnatal Depression Score (EPDS) [14] of 12 or more (proxy for PND) at weeks 12/13, 21, 32 of pregnancy and weeks 4 and 12 post-delivery using the baseline (week 4–10 of pregnancy) survey responses as predictors.

### Source of data

**Fig.1:**
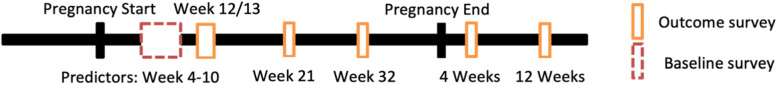
Survey timeline used to capture the data for this study

This study was a prospective study containing self-reported survey data measured at multiple time points during and after pregnancy. The survey, which was previously published [15], was conducted for the purpose of developing risk models. The survey was advertised to women interacting with the BabyCenter website, an educational and informational website for moms, spouses and partners. Women were enrolled into the study between week 4 to week 10 of their pregnancy. The participants filled in a survey asking them about their lifestyle, social economics, health history and five psychiatric measures at enrolment (baseline). The participants were then followed longitudinally throughout pregnancy and after delivery and a survey containing the EPDS was given to participants five times after baseline (during each trimester and two times after delivery), see Fig. 1. The first EPDS score post baseline used in this study was given at weeks 12 or 13 depending on the baseline enrolment week. This was administered between 3 and 8 weeks after baseline. Two more EPDS scores during pregnancy (trimester 2 at week 21 and trimester 3 at week 32) and two more EPDS scores after delivery (week 4 and week 12 post-delivery) were also included in this study.

### Participants

The participants of this study were women who were active on the BabyCenter website, between August 25 to September 19, 2016, and consented for participation. Eligibility criteria were that the participant must be 4–10 weeks pregnant at enrolment. Participants were excluded if any of the following were true: male gender, location outside the US, age less than 18, or participating in other studies. The sample of pregnant women enrolled into the study appear to be representative of the US adult population [15].

Participants were paid for each survey completed and could have received up to $180 if all surveys were completed. In addition, participants were included into a $1000 sweepstake and the number of entries per person depended on how many surveys they completed.

### Outcome

We predicted perinatal depression during five different time points: each trimester and at two periods after delivery. Perinatal depression at a given time period was defined as an EPDS score (the most recent during the time period) of 12 or more.

The EPDS is a measure that has been developed to assess the risk of perinatal depression but is not a clinical diagnosis of depression. An EPDS of 14 or more is often used as a cut off to divide into high risk and low risk of depressive illness. In this study we used an EPDS score of 12 or more as a proxy for depression as the self-harm question was missing from the self-reported survey used in this study due to ethical considerations. It has been shown that the EPDS score identified major depression with a sensitivity of 88%, a specificity of 92.5% and a positive predictive value of 35.1% [16].

### Predictors

We used the self-reported baseline survey response to construct predictors. The baseline survey included 180 different questions on lifestyle, social economics, health history, various psychiatric measures (i.e., state-trait anxiety inventory, generalized anxiety disorder (GAD) [17], PROMIS emotional support (PRES) [18] and perceived stress scale (PSS) [19] and the baseline EPDS questions excluding the self-harm question). The GAD scale contains 8 questions, the EPDS (less the suicide question) contains 9 questions and the PRES contains 4 questions. Complete details about the survey, including all the baseline survey questions, has been published [15].

The baseline predictors are a combination of ordinal variables, binary indicator variables and category variables. Every participant filled out the baseline survey. The baseline psychiatric measures (EPDS, GAD, state-trait anxiety inventory, PRES and PSS) were answered fully by participants but questions on lifestyle, social economics and health history were occasionally missed.

### Sample size

5,028 BabyCenter users showed interest in completing the survey. 3,471 were excluded due to pregnancy outside weeks of interest (2,186), not completing the screening Sect. (557), not being pregnant (317), participating in other research (190), age less than 18 (151), located outside the US (75) and being male (55). This left 1,557 qualified to participate and 1,179 (76%) completed the baseline survey. Eight hundred and fifty-eight (858) of these participants were 4–10 weeks pregnant and 321 were 28 to 33 weeks pregnant. This study only used the 858 participants at 4–10 weeks of their pregnancy who completed the baseline survey. 554, 528, 555, 469 and 515 of these participants filled in the EPDS survey during trimester 1 (week 12 or 13), trimester 2 (week 21), trimester 3 (week 32), week 4 post-delivery and week 12 post-delivery, respectively.

### Missing data

Predictors: All participants filled out the baseline survey, but some non-psychiatric measures questions were optional resulting in some missing data. To address this, we excluded 16 baseline non-psychiatric measure variables due to insufficient responses (a binary indicator where nobody selected ‘Yes’ or an ordinal/category response that was answered by < 50% of participants). For the remaining ordinal and category variables we used mode imputation when values were missing. For the indicator variables, participants had to select ‘Yes’ otherwise the response defaulted to ‘No’. This means that if a participant did not answer the question, she would have a ‘No’ response rather than a missing value. Therefore, the response ‘No’ means they did not have the variable, or they did not respond.

Outcome: The follow-up EPDS surveys were not compulsory and were missed by a significant number of participants. We excluded patients from the data used to train and evaluate each model if they did not complete the EPDS survey at the specific time point being predicted. We investigated the differences in baseline responses between patients who were excluded and those used in each model development to quantify how excluding patients without the outcome may compromise generalizability of the models.

### Statistical analysis methods

We investigated three different predictor sets:

- i) [GAD/EPDS] Baseline GAD and EPDS scale questions
- ii) [GAD/EPDS/PRES] Baseline GAD, EPDS and the PRES scale questions
- iii) [All 164 predictors] Baseline non-scale questions (e.g., health history, demographics, lifestyle, partner’s mental health) plus the GAD, EPDS and the PRES questions

For each outcome and predictor set we trained a gradient boosting machine [20]. A gradient boosting machine was chosen due to the psychiatric measure variables often being ordinal and tree-based models can account for non-linear relationships. We split the data into 80% training and 20% testing sets. We used ten-fold cross validation repeated ten times on the training data to identify the optimal hyper-parameters and then trained a final model with the optimal hyper-parameters using all the training data. We internally evaluated the model on the 20% test set by calculating the discriminative ability using the area under the receiver operating characteristic curve (AUC). The sensitivity (percentage of the actual depressed patients that are predicted to be depressed) and positive predictive values (the percentage of actual depressed people in the patients predicted to be depressed) are also presented at various thresholds. We repeated the above process 10 times with different train/test splits to calculate confidence intervals for the performance estimates.

To calculate the predictor importance, we used SHapley Additive explanation (SHAP) [21]. SHAP uses a game theory approach to estimate the impact that each predictor has on participants’ predicted risks. This can provide predictor important globally across all participants as well as locally for a specific participant.

## Results

### Participants

858 women were enrolled into the study and filled in the baseline survey. 554, 528, 555, 469 and 515 of these women filled in the EPDS survey during trimester 1, trimester 2, trimester 3, week 4 post-delivery and week 12 post-delivery, respectively. To quantify whether the women who completed the EPDS survey at each follow-up time-period were different at baseline from those who did not, we developed models to predict who would participate in each follow-up EPDS survey using all baseline predictors and ten-fold cross validation repeated 10 times. The ability to predict participation was moderately weak with cross-validation AUCs ranging between 0.63–0.66 when using all variables and between 0.55–0.57 when using the GAD/EPDS/PRES variables across the time periods. This suggests there are some small baseline differences in those who drop out and those who do not. Some of the baseline responses associated with participation were being married, ethnicity, not feeling upset at baseline, using a desktop computer, history of yoga, partner not having any existing mental health issues, diet, sleep issues and income.

The full details of the characteristics of the missing vs present people at each follow-up timepoint are presented in Additional file 1.

### Gradient boosting machine models

The number of participants  and outcome sizes are presented in Table 1.

**Table 1:** The data sizes and outcome count for the different time-periods investigated

Follow-up Period	Participant Count	Outcome Count	Outcome %
Trimester 1	554	116	20.9
Trimester 2	528	111	20.0
Trimester 3	555	140	25.2
After deliver 1	469	77	16.4
After deliver 2	515	89	17.3

**Fig. 2:**
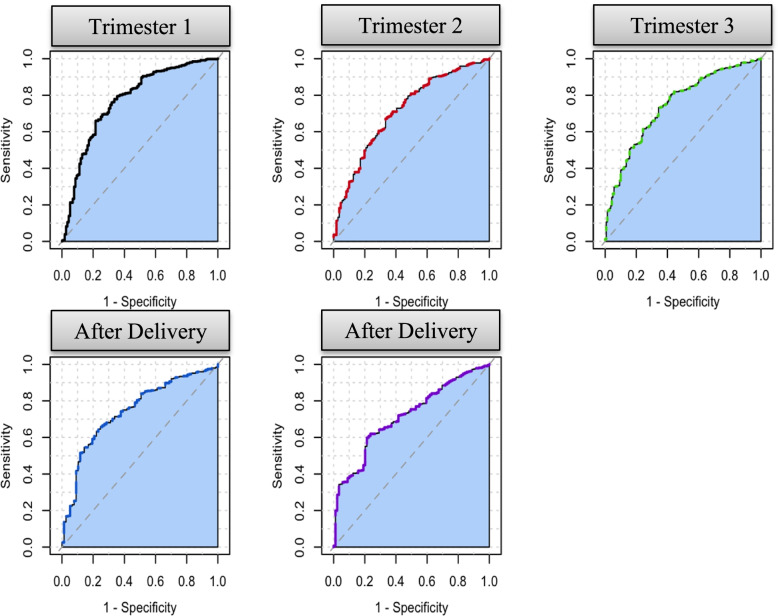
ROC plots for the five gradient boosting machine models using EDPS/GAD/PRES predictors

**Fig. 3:**
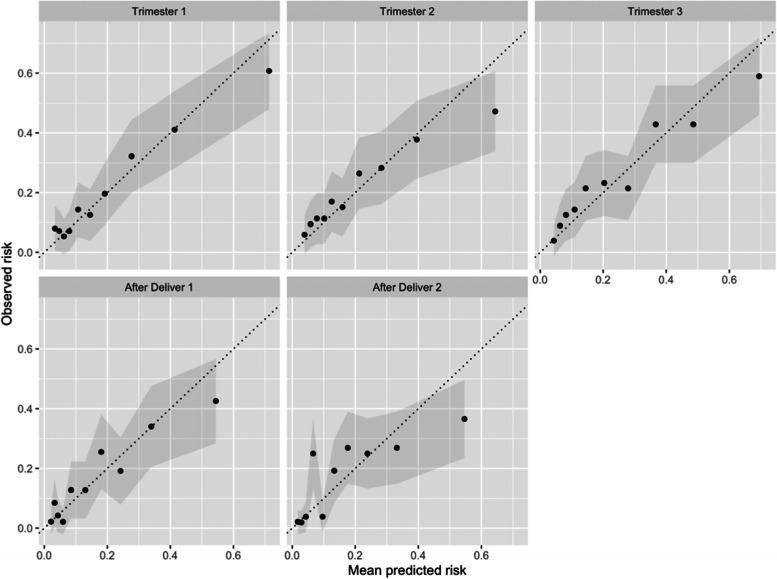
calibration plots for the five gradient boosting machine models using EPDS/GAD/PRES predictors. The validation set was partitioned into ten groups based on predicted risk. Each dot represents one of the ten groups. The mean risk within the group is plotted against the observed risk. If the dot falls on the diagonal line, then the predicted risk on average matches the observed risk, indicating excellent calibration. The shaded region is the confidence interval

The baseline characteristics of those who have an EPDS score of 12 or greater during each time period and those who do not are presented in Additional file 2. The performance of the models for each EPDS follow-up time period and predictor set are presented in Table 2. Receiver Operating Characteristic (ROC) curves and calibration plots for the models using the 21 GAD/EPDS/PRES predictors are presented in Figs. 2 and 3. We investigated logistic regression and decision tree as alternative classifiers, but the gradient boosting machine performances were generally better, see Additional file 3.

**Table 2:** The discriminative performance of the models using different predictor sets

Predictor Set	Predictor Count	AUC (95% CI)				
		Trimester 1	Trimester 2	Trimester 3	After delivery 1	After delivery 2
GAD/EPDS	17	0.78 (0.75–0.80)	0.69 (0.65–0.74)	0.73 (0.69–0.78)	0.71 (0.67–0.76)	0.69 (0.64–0.73)
**GAD/EPDS/PRES**	**21**	**0.77 (0.74–0.80)**	**0.69 (0.65–0.73)**	**0.75 (0.71–0.79)**	**0.72 (0.67–0.78)**	**0.71 (0.66–0.76)**
All Predictors	164	0.75 (0.72–0.78)	0.69 (0.65–0.72)	0.74 (0.71–0.78)	0.73 (0.68–0.77)	0.70 (0.64–0.77)

The SHAP variable importance for each model using the GAD/EPDS/PRES predictor set is presented in Additional file 4.

**Fig. 4:**
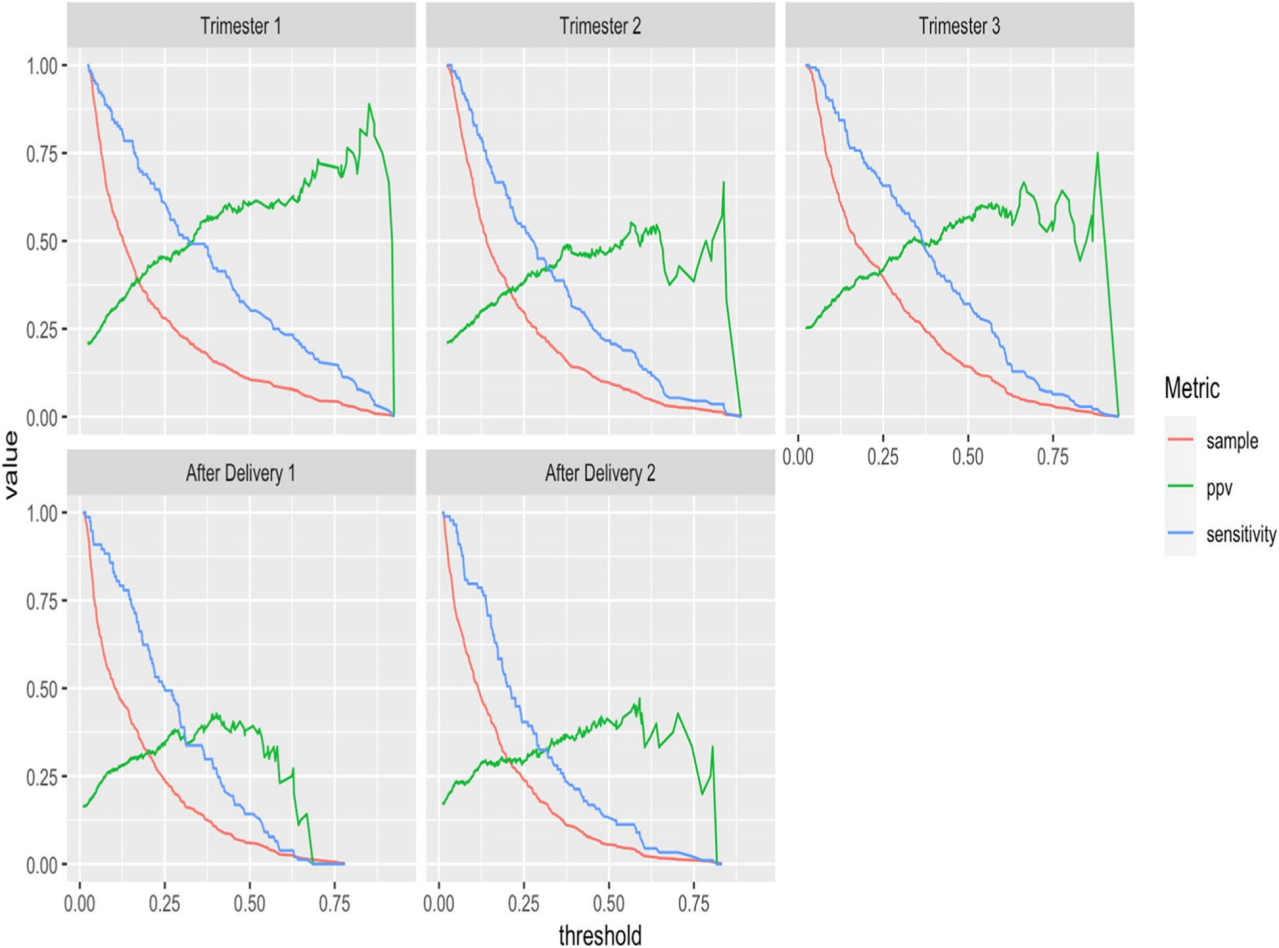
The probability threshold plot [22] showing the sample (the proportion of the population who is classified ‘will have depression’ by the model at the given threshold), the PPV (the proportion of people classified ‘will have depression’ who truly have a high EPDS score) and sensitivity (the proportion of people with a high EPDS who are classified ‘will have depression’ by the model) across all possible decision thresholds

The positive predictive value (PPV) of the GAD/EPDS/PRES models at various sensitivity cut points are presented in Table 3 and the values for all possible decision thresholds are presented in Fig. 4. The decision threshold is the value such that pregnant women with a predicted risk greater than or equal to the threshold are classified as ‘will have depression’ by the model.

**Table 3:** The sensitivity, positive predictive value (PPV) at various decision threshold for the five models using the EPDS/GAD/PRES predictors. The decision threshold is the value at which women predicted to have a risk greater or equal to are classified as ‘will have depression’ by the prediction model. When implementing a model for decision support, a suitable decision threshold needs to be determined based on the required sensitivity/PPV

Sensitivity	Trimester 1			Trimester 2			Trimester 3			After delivery 1			After delivery 2		
	Threshold	% > = threshold	PPV (%)	Threshold	% > = threshold	PPV (%)	Threshold	% > = threshold	PPV (%)	Threshold	% > = threshold	PPV (%)	Threshold	% > = threshold	PPV (%)
10%	0.80	2.9	75.0	0.65	4.0	52.4	0.70	4.1	60.9	0.54	5.1	33.3	0.58	3.9	45.0
20%	0.66	6.1	67.6	0.54	8.3	50.0	0.60	8.5	59.6	0.45	7.9	40.5	0.42	9.3	37.5
30%	0.52	10.3	61.4	0.43	13.4	46.5	0.51	13.2	57.5	0.39	11.5	42.6	0.33	14.8	35.5
40%	0.44	13.7	60.5	0.34	19.3	43.1	0.43	18.7	53.8	0.30	18.3	36.0	0.26	22.3	31.3
50%	0.32	21.3	49.2	0.27	25.8	41.1	0.37	25.6	49.3	0.25	24.1	34.5	0.21	28.9	29.5
60%	0.25	28.0	45.2	0.21	35.6	35.6	0.30	32.8	46.2	0.21	30.7	31.9	0.17	36.1	28.5
70%	0.18	36.1	40.5	0.15	46.0	32.0	0.22	43.2	40.8	0.17	38.2	30.2	0.15	41.6	29.0
80%	0.13	49.8	33.7	0.12	56.6	29.8	0.14	55.1	36.6	0.12	47.3	27.9	0.11	50.9	27.1
90%	0.07	69.0	27.2	0.08	72.2	26.2	0.10	68.3	33.2	0.07	59.3	24.8	0.07	66.2	23.5

The gradient boosting machine hyper-parameter grid search settings used in this study are available in Additional file 5.

The models are available via an R package saved to a GitHub repository (https://github.com/jreps/PND). This is to enable other researcher to validate the models, not to enable clinical implementation.

## Discussion & conclusion

### Interpretation

We investigated models that predict depression (using EPDS >  = 12 as a proxy) at five time periods during and after pregnancy using survey responses during weeks 4–10 of pregnancy as predictors. In the first two trimesters approximately 20% of the women surveyed had an EPDS >  = 12, this increased to 25% in the final trimester and then decreased to 16%-17% following delivery.

We developed models using three predictor sets: i) 17 questions from the EPDS and GAD scales, ii) 21 questions from the EPDS, GAD and PRES scales and iii) 164 questions including additional psychiatric scales, demographics, lifestyle, medical history and partner mental health questions. The results show that the performance was similar for all three predictor sets, with models tending to overfit when all 164 variables were used due to the small data size. Including the PRES scale questions tended to improve the prediction of depression after delivery, although the performance was not significantly better than the models using EPDS/GAD only.

The SHAP results indicate that crying early in pregnancy is a key predictor of high EPDS scores during pregnancy. In general, showing signs of depression/anxiety at week 4–10 was predictive of a high EPDS throughout pregnancy. Baseline predictors of a high EPDS after delivery were anxiety (worrying, nervousness and anxiety), difficulty sleeping and feeling afraid. Having somebody who makes you feel appreciated appears to be associated with lower EPDS score after pregnancy, however causality was not investigated in this study.

Focusing on the models developed using the 21 questions from the EPDS, GAD and PRES scales, the models AUC performance across the time periods ranged between low to middle 70 s, with trimester 1 being the easier to predict. This is expected, as trimester 1 was closest in time to the baseline survey. The calibration plots indicate reasonable calibration, although the models appear to slightly over-estimate risk for the highest risk groups. When predicting an EPDS >  = 12 after delivery during weeks 4 and 12, the calibration plots show there is a group of women who are assigned a risk around 10% but approximately 25% of these women had an EPDS >  = 12. This may be due to the model using variables early in pregnancy, which may be insufficient to identify these women as high risk after delivery.

In general, our models performed similarly compared to existing models when matched to a similar prediction time point. Our model using survey responses at week 4–10 of pregnancy to predict postpartum depression 12 weeks after delivery (predicting depression ~ 42 weeks in the future) had a 35.5% PPV at 30% sensitivity. This is comparable to the Brisbane Postnatal Depression Index that uses antenatal and postnatal variables to predict 16-week postpartum depression (predicting depression ~ 16 weeks in the future) that had a 40% PPV at 36.3% sensitivity. Our model using survey responses at week 4–10 of pregnancy to predict postpartum depression 4 weeks after delivery (predicting depression ~ 34 weeks in the future) had a PPV of 42.6% at 30% sensitivity. This is slightly better than an existing model that uses variables collected during the 3^rd^ trimester to predict postpartum depression 6–8 weeks postpartum (predicting depression between 7–20 weeks in the future) that had a PPV of 35% at 33% sensitivity [11]. However, we developed models for multiple time periods including during early pregnancy, which is rarely predicted. Our model was also unique because it only used variables that were collected early in trimester 1, making it applicable at an earlier point than existing models.

To use the models a patient would need to be asked only 21 questions at week 4 to 10 of their pregnancy. These 21 items could easily be assessed via online survey, phone or tablet to determine risk at the different time-points. If using the models for decision making, we provide the PPV and sensitivities for nine different thresholds, see Table 3. The desirable threshold will depend on how the models will be used. For example, if the model is used to identify patients who may benefit from additional education or depression screening, then a high sensitivity may be preferred at the cost of having a higher false positive rate (lower PPV). Alternatively, if the models are used to identify patients who may benefit from some restricted intervention, then a high PPV may be more desirable.

### Implications

Our models can be implemented early in pregnancy (week 4–10) by asking women to complete 3 common psychological scales to calculate a personal risk of developing depression at different time points during and after delivery. If a patient is assigned into the higher risk groups, then the care provider may wish to educate the patient more about perinatal depression and the symptoms or set up screening appointments during the time periods they are at high risk.

Currently, screening for depression is rarely done during and after pregnancy. A possible intervention for early detection of depression during and after pregnancy is for a healthcare worker to schedule regular depression screening visits for pregnant women. This intervention would be constrained by the availability of suitable healthcare workers who can perform the screenings. It is probably infeasible to screen all pregnant women multiple times during and after pregnancy. But our model could be used to target a small subset of these pregnant women to screen. For example, our models could be applied by performing the 21-question survey to each newly pregnant women to identify which, if any, of the future time points the women may be at risk of depression. Those predicted to be high risk during trimester 1 could have a screening meeting during trimester 1 planned and this could be repeated for each of the five time periods investigated in this study. The number of screening visits will depend on the availability of staff. If it is only possible to screen 10% of pregnant women during trimester 1, then the threshold in Table 3 that results in ~ 10% of patients being deemed high risk for trimester 1 could be used. Using our model, this would be a decision threshold of 0.52 resulting in a sensitivity of 30% and PPV of 61.4%. Therefore, ~ 30% of women who may have new depression during trimester 1 could be identified and receive treatment during trimester 1, but this would only require screening 10% of pregnant women. If it is possible to screen more pregnant women, then the decision threshold could be lowered, helping to improve the sensitivity.

### Limitations

The main limitation of this study is that a high percentage of women dropped out after the baseline survey. This may impact the generalizability of the models to the general population as the dropout may be associated with having or developing depression. We were unable to find any strong predictor of dropout using baseline variables. This suggests that the model may be generalizable, however it is import for these models to be externally validated to confirm this. Based on Grade and Assess Predictive tools (GRASP) guidelines, the models need to be externally validated and also prospectively tested in any clinically setting they may be applied before the true performance is known [23]. Another limitation is that the EPDS score was used as a proxy for depression and the EPDS score is not a clinical diagnosis. In future work it would be useful to validate the model on data that has a clinical definition of perinatal depression as the outcome.

As we used gradient boosting machines, the models are hard to interpret. We used SHAP to provide variable importance plots to show which variables had more impact in the risk predictions. SHAP can also be used when using the online calculator to understand what contributed to the high risk. The SHAP values for visualizing the importance of each variable in the final models are provided as a weak form of trust by showing the important variables intuitively make sense. However, there are numerous publications showing the limitations of trying to interpret black box models [24] and we do not recommend readers overinterpreting the SHAP results. Trust in a model can only be gained by prospectively evaluating the model in the clinically settings it will be applied. This is an important area of future work.

A key strength of this study is that it used a prospective cohort design, but this resulted in having a smaller dataset of around 500–600 patients and outcome counts ranging between 77–140. The low outcome count limited the complexity of the models, so more discriminative models may be possible to learn with more data. It also decreases the confidence in the model performance estimates, leading to wider confidence intervals.

## Conclusion

In this paper we developed five models that only require asking 21 questions at week 4–10 of pregnancy and can be used to predict whether a patient is at high or low risk of experiencing depression during each trimester and during two time periods after delivery. The models could be used to identify patients who would benefit from certain interventions, such as additional education about depression or more regular check-ups and depression screening. In future work it is important to examine the generalizability of the models by externally validating them on new patients or prospectively evaluating the models. It would also be beneficial to test the models’ performances when using clinically defined depression as the outcome.

## Supplementary Information

**Additional file 1.** Characteristics of missing outcomes. A word document containing a table with the mean values for each baseline survey question for those who completed each outcome survey vs those who did not (missing women).

**Additional file 2.** Characteristics of people with and without the outcomes. A word document containing a table with the mean values for each of the baseline survey for EPDS/GAD/PRES for those who had an EPDS < 12 vs an EPDS >= 12 for each outcome survey.

**Additional file 3.** Comparison of machine learning methods. A word document containing the details of model tuning for the gradient boosting machine, logistic regression, and decision tree.

**Additional file 4.** SHAP results. A word document with the SHAP results for each outcome.

**Additional file 5.** Hyper-parameter search for gradient boosting machine. A word document containing the hyper-parameter grid search values for the gradient boosting machine model. 

## Data Availability

The data that support the findings cannot be shared due to privacy/ethical restrictions. The models developed in this study are available from https://github.com/jreps/PND.

## Abbreviations

A1: After delivery 1
A2: After delivery 2
AUC: Area under the receiver operating characteristic curve
EPDS: Edinburgh Postnatal Depression Score
GAD: Generalized anxiety disorder
GRASP: Grade and Assess Predictive tools
PND: Perinatal depression
PPV: Positive predictive value
PRES: PROMIS emotional support
PSS: Perceived stress scale
ROC: Receiver Operating Characteristic
SHAP: SHapley Additive explanation
T1: Trimester 1
T2: Trimester 2
T3: Trimester 3
