# Supplementary material for: Development of multivariable models to predict perinatal depression before and after delivery using patient reported survey responses at weeks 4–10 of pregnancy
Source: BMC Pregnancy Childbirth. 2022 May 26;22:442. doi: 10.1186/s12884-022-04741-9 (PMC9137134; doi:10.1186/s12884-022-04741-9)
Supplement: Supplementary file 1 — Additional file 1. Characteristics of missing outcomes. A word document containing a table with the mean values for each baseline survey question for those who completed each outcome survey vs those who did not (missing women). [file 12884_2022_4741_MOESM1_ESM.docx]

Additional file 1: Characteristics of missing outcomes

|  | Mean value Trimester 1 | | Mean value Trimester 2 | | Mean value Trimester 3 | | Mean value After 1 | | Mean value After 2 | |
| --- | --- | --- | --- | --- | --- | --- | --- | --- | --- | --- |
| Variable: Value | Present | Missing | Present | Missing | Present | Missing | present | Missing | present | Missing |
| I have been able to laugh and see the funny side of things.: 1 | 0.79 | 0.75 | 0.78 | 0.77 | 0.78 | 0.76 | 0.79 | 0.76 | 0.79 | 0.76 |
| I have been able to laugh and see the funny side of things.: 2 | 0.17 | 0.21 | 0.18 | 0.19 | 0.18 | 0.2 | 0.18 | 0.2 | 0.18 | 0.2 |
| I have been able to laugh and see the funny side of things.: 3 | 0.03 | 0.02 | 0.03 | 0.02 | 0.03 | 0.02 | 0.02 | 0.03 | 0.02 | 0.03 |
| I have been able to laugh and see the funny side of things.: 4 | 0.01 | 0.01 | 0.01 | 0.01 | 0.01 | 0.01 | 0.01 | 0.01 | 0.01 | 0.01 |
| I have looked forward with enjoyment to things.: 1 | 0.75 | 0.69 | 0.75 | 0.7 | 0.76 | 0.68 | 0.77 | 0.68 | 0.77 | 0.67 |
| I have looked forward with enjoyment to things.: 2 | 0.21 | 0.25 | 0.2 | 0.25 | 0.2 | 0.26 | 0.19 | 0.26 | 0.2 | 0.26 |
| I have looked forward with enjoyment to things.: 3 | 0.04 | 0.05 | 0.04 | 0.04 | 0.03 | 0.05 | 0.04 | 0.05 | 0.03 | 0.05 |
| I have looked forward with enjoyment to things.: 4 | 0.01 | 0.01 | 0.01 | 0.01 | 0.01 | 0.01 | 0 | 0.02 | 0 | 0.02 |
| I have blamed myself unnecessarily when things went wrong.: 1 | 0.24 | 0.2 | 0.24 | 0.21 | 0.24 | 0.21 | 0.24 | 0.21 | 0.24 | 0.2 |
| I have blamed myself unnecessarily when things went wrong.: 2 | 0.41 | 0.39 | 0.41 | 0.38 | 0.42 | 0.37 | 0.43 | 0.37 | 0.42 | 0.38 |
| I have blamed myself unnecessarily when things went wrong.: 3 | 0.3 | 0.32 | 0.3 | 0.33 | 0.29 | 0.35 | 0.28 | 0.34 | 0.29 | 0.33 |
| I have blamed myself unnecessarily when things went wrong.: 4 | 0.05 | 0.09 | 0.05 | 0.08 | 0.06 | 0.08 | 0.05 | 0.08 | 0.05 | 0.09 |
| I have been anxious or worried for no good reason.: 1 | 0.2 | 0.18 | 0.2 | 0.18 | 0.2 | 0.17 | 0.21 | 0.17 | 0.21 | 0.17 |
| I have been anxious or worried for no good reason.: 2 | 0.26 | 0.26 | 0.26 | 0.25 | 0.27 | 0.24 | 0.28 | 0.24 | 0.26 | 0.25 |
| I have been anxious or worried for no good reason.: 3 | 0.47 | 0.48 | 0.46 | 0.49 | 0.46 | 0.5 | 0.46 | 0.49 | 0.47 | 0.48 |
| I have been anxious or worried for no good reason.: 4 | 0.07 | 0.08 | 0.07 | 0.08 | 0.07 | 0.08 | 0.06 | 0.1 | 0.06 | 0.1 |
| I have felt scared or panicky for no very good reason.: 1 | 0.34 | 0.31 | 0.33 | 0.33 | 0.34 | 0.31 | 0.34 | 0.31 | 0.33 | 0.32 |
| I have felt scared or panicky for no very good reason.: 2 | 0.31 | 0.3 | 0.3 | 0.31 | 0.31 | 0.29 | 0.32 | 0.3 | 0.32 | 0.29 |
| I have felt scared or panicky for no very good reason.: 3 | 0.32 | 0.34 | 0.33 | 0.32 | 0.31 | 0.35 | 0.3 | 0.35 | 0.31 | 0.34 |
| I have felt scared or panicky for no very good reason.: 4 | 0.03 | 0.06 | 0.04 | 0.05 | 0.04 | 0.05 | 0.04 | 0.04 | 0.04 | 0.05 |
| Things have been getting on top of me.: 1 | 0.24 | 0.21 | 0.24 | 0.21 | 0.25 | 0.19 | 0.24 | 0.21 | 0.24 | 0.2 |
| Things have been getting on top of me.: 2 | 0.48 | 0.44 | 0.47 | 0.45 | 0.47 | 0.46 | 0.48 | 0.46 | 0.47 | 0.45 |
| Things have been getting on top of me.: 3 | 0.24 | 0.3 | 0.26 | 0.27 | 0.26 | 0.27 | 0.25 | 0.28 | 0.26 | 0.27 |
| Things have been getting on top of me.: 4 | 0.04 | 0.06 | 0.03 | 0.07 | 0.03 | 0.08 | 0.03 | 0.06 | 0.03 | 0.07 |
| I have been so unhappy that I have had difficulty sleeping.: 1 | 0.68 | 0.57 | 0.68 | 0.58 | 0.67 | 0.57 | 0.69 | 0.57 | 0.68 | 0.57 |
| I have been so unhappy that I have had difficulty sleeping.: 2 | 0.2 | 0.26 | 0.21 | 0.24 | 0.2 | 0.26 | 0.2 | 0.25 | 0.21 | 0.24 |
| I have been so unhappy that I have had difficulty sleeping.: 3 | 0.09 | 0.14 | 0.08 | 0.15 | 0.1 | 0.13 | 0.09 | 0.14 | 0.08 | 0.15 |
| I have been so unhappy that I have had difficulty sleeping.: 4 | 0.03 | 0.03 | 0.03 | 0.04 | 0.03 | 0.04 | 0.03 | 0.04 | 0.03 | 0.04 |
| I have felt sad or miserable.: 1 | 0.53 | 0.49 | 0.54 | 0.49 | 0.54 | 0.48 | 0.55 | 0.48 | 0.54 | 0.48 |
| I have felt sad or miserable.: 2 | 0.29 | 0.24 | 0.29 | 0.24 | 0.29 | 0.24 | 0.3 | 0.23 | 0.3 | 0.22 |
| I have felt sad or miserable.: 3 | 0.16 | 0.22 | 0.15 | 0.22 | 0.15 | 0.23 | 0.13 | 0.24 | 0.14 | 0.23 |
| I have felt sad or miserable.: 4 | 0.02 | 0.05 | 0.02 | 0.05 | 0.02 | 0.06 | 0.01 | 0.05 | 0.02 | 0.06 |
| I have been so unhappy that I have been crying.: 1 | 0.63 | 0.54 | 0.63 | 0.54 | 0.64 | 0.51 | 0.65 | 0.53 | 0.64 | 0.53 |
| I have been so unhappy that I have been crying.: 2 | 0.32 | 0.37 | 0.31 | 0.37 | 0.31 | 0.38 | 0.3 | 0.38 | 0.31 | 0.37 |
| I have been so unhappy that I have been crying.: 3 | 0.05 | 0.08 | 0.05 | 0.07 | 0.04 | 0.08 | 0.04 | 0.07 | 0.04 | 0.08 |
| I have been so unhappy that I have been crying.: 4 | 0.01 | 0.02 | 0.01 | 0.02 | 0.01 | 0.02 | 0.01 | 0.02 | 0.01 | 0.02 |
| Thinking about how you‚Äôve been feeling more recently, over the last 2 weeks, how often have you b...-Feeling nervous, anxious, or on edge: 1 | 0.41 | 0.41 | 0.4 | 0.43 | 0.4 | 0.43 | 0.42 | 0.39 | 0.41 | 0.41 |
| Thinking about how you‚Äôve been feeling more recently, over the last 2 weeks, how often have you b...-Feeling nervous, anxious, or on edge: 2 | 0.39 | 0.36 | 0.4 | 0.33 | 0.4 | 0.33 | 0.39 | 0.36 | 0.39 | 0.36 |
| Thinking about how you‚Äôve been feeling more recently, over the last 2 weeks, how often have you b...-Feeling nervous, anxious, or on edge: 3 | 0.14 | 0.14 | 0.12 | 0.16 | 0.13 | 0.16 | 0.12 | 0.16 | 0.13 | 0.15 |
| Thinking about how you‚Äôve been feeling more recently, over the last 2 weeks, how often have you b...-Feeling nervous, anxious, or on edge: 4 | 0.07 | 0.09 | 0.07 | 0.08 | 0.07 | 0.08 | 0.07 | 0.08 | 0.07 | 0.08 |
| Thinking about how you‚Äôve been feeling more recently, over the last 2 weeks, how often have you b...-Not being able to stop or control worrying: 1 | 0.54 | 0.52 | 0.55 | 0.51 | 0.54 | 0.51 | 0.57 | 0.49 | 0.56 | 0.49 |
| Thinking about how you‚Äôve been feeling more recently, over the last 2 weeks, how often have you b...-Not being able to stop or control worrying: 2 | 0.29 | 0.3 | 0.29 | 0.28 | 0.29 | 0.29 | 0.28 | 0.31 | 0.28 | 0.3 |
| Thinking about how you‚Äôve been feeling more recently, over the last 2 weeks, how often have you b...-Not being able to stop or control worrying: 3 | 0.12 | 0.11 | 0.1 | 0.14 | 0.12 | 0.12 | 0.1 | 0.14 | 0.11 | 0.13 |
| Thinking about how you‚Äôve been feeling more recently, over the last 2 weeks, how often have you b...-Not being able to stop or control worrying: 4 | 0.05 | 0.08 | 0.05 | 0.07 | 0.05 | 0.08 | 0.05 | 0.07 | 0.05 | 0.07 |
| Thinking about how you‚Äôve been feeling more recently, over the last 2 weeks, how often have you b...-Worrying too much about different things: 1 | 0.42 | 0.38 | 0.43 | 0.37 | 0.43 | 0.37 | 0.45 | 0.36 | 0.43 | 0.36 |
| Thinking about how you‚Äôve been feeling more recently, over the last 2 weeks, how often have you b...-Worrying too much about different things: 2 | 0.38 | 0.44 | 0.39 | 0.42 | 0.39 | 0.43 | 0.37 | 0.43 | 0.38 | 0.43 |
| Thinking about how you‚Äôve been feeling more recently, over the last 2 weeks, how often have you b...-Worrying too much about different things: 3 | 0.13 | 0.11 | 0.12 | 0.12 | 0.13 | 0.11 | 0.12 | 0.12 | 0.13 | 0.12 |
| Thinking about how you‚Äôve been feeling more recently, over the last 2 weeks, how often have you b...-Worrying too much about different things: 4 | 0.07 | 0.08 | 0.06 | 0.08 | 0.06 | 0.09 | 0.06 | 0.08 | 0.06 | 0.09 |
| Thinking about how you‚Äôve been feeling more recently, over the last 2 weeks, how often have you b...-Trouble relaxing: 1 | 0.45 | 0.43 | 0.46 | 0.42 | 0.46 | 0.42 | 0.46 | 0.42 | 0.45 | 0.43 |
| Thinking about how you‚Äôve been feeling more recently, over the last 2 weeks, how often have you b...-Trouble relaxing: 2 | 0.39 | 0.36 | 0.37 | 0.39 | 0.38 | 0.38 | 0.37 | 0.38 | 0.38 | 0.38 |
| Thinking about how you‚Äôve been feeling more recently, over the last 2 weeks, how often have you b...-Trouble relaxing: 3 | 0.1 | 0.14 | 0.12 | 0.12 | 0.11 | 0.13 | 0.11 | 0.12 | 0.12 | 0.12 |
| Thinking about how you‚Äôve been feeling more recently, over the last 2 weeks, how often have you b...-Trouble relaxing: 4 | 0.06 | 0.07 | 0.06 | 0.07 | 0.06 | 0.08 | 0.06 | 0.07 | 0.05 | 0.08 |
| Thinking about how you‚Äôve been feeling more recently, over the last 2 weeks, how often have you b...-Being so restless that it's hard to sit still: 1 | 0.68 | 0.62 | 0.68 | 0.62 | 0.69 | 0.6 | 0.69 | 0.62 | 0.69 | 0.62 |
| Thinking about how you‚Äôve been feeling more recently, over the last 2 weeks, how often have you b...-Being so restless that it's hard to sit still: 2 | 0.2 | 0.22 | 0.2 | 0.21 | 0.2 | 0.21 | 0.19 | 0.22 | 0.2 | 0.22 |
| Thinking about how you‚Äôve been feeling more recently, over the last 2 weeks, how often have you b...-Being so restless that it's hard to sit still: 3 | 0.08 | 0.11 | 0.08 | 0.12 | 0.07 | 0.14 | 0.08 | 0.11 | 0.08 | 0.12 |
| Thinking about how you‚Äôve been feeling more recently, over the last 2 weeks, how often have you b...-Being so restless that it's hard to sit still: 4 | 0.04 | 0.05 | 0.04 | 0.05 | 0.04 | 0.05 | 0.04 | 0.05 | 0.04 | 0.05 |
| Thinking about how you‚Äôve been feeling more recently, over the last 2 weeks, how often have you b...-Becoming easily annoyed or irritable: 1 | 0.22 | 0.26 | 0.23 | 0.25 | 0.23 | 0.25 | 0.23 | 0.24 | 0.23 | 0.24 |
| Thinking about how you‚Äôve been feeling more recently, over the last 2 weeks, how often have you b...-Becoming easily annoyed or irritable: 2 | 0.47 | 0.36 | 0.47 | 0.36 | 0.46 | 0.36 | 0.46 | 0.39 | 0.46 | 0.38 |
| Thinking about how you‚Äôve been feeling more recently, over the last 2 weeks, how often have you b...-Becoming easily annoyed or irritable: 3 | 0.19 | 0.2 | 0.19 | 0.2 | 0.19 | 0.2 | 0.2 | 0.19 | 0.2 | 0.19 |
| Thinking about how you‚Äôve been feeling more recently, over the last 2 weeks, how often have you b...-Becoming easily annoyed or irritable: 4 | 0.12 | 0.19 | 0.12 | 0.19 | 0.12 | 0.19 | 0.11 | 0.19 | 0.11 | 0.19 |
| Thinking about how you‚Äôve been feeling more recently, over the last 2 weeks, how often have you b...-Feeling afraid as if something awful might happen: 1 | 0.51 | 0.47 | 0.5 | 0.48 | 0.49 | 0.5 | 0.51 | 0.48 | 0.5 | 0.49 |
| Thinking about how you‚Äôve been feeling more recently, over the last 2 weeks, how often have you b...-Feeling afraid as if something awful might happen: 2 | 0.3 | 0.31 | 0.3 | 0.3 | 0.31 | 0.29 | 0.3 | 0.31 | 0.31 | 0.29 |
| Thinking about how you‚Äôve been feeling more recently, over the last 2 weeks, how often have you b...-Feeling afraid as if something awful might happen: 3 | 0.11 | 0.12 | 0.11 | 0.12 | 0.13 | 0.1 | 0.12 | 0.11 | 0.12 | 0.12 |
| Thinking about how you‚Äôve been feeling more recently, over the last 2 weeks, how often have you b...-Feeling afraid as if something awful might happen: 4 | 0.08 | 0.1 | 0.09 | 0.09 | 0.08 | 0.11 | 0.07 | 0.11 | 0.08 | 0.11 |
| Thinking about how you‚Äôve been feeling more recently, over the last 2 weeks, how often have you b...-Feeling I might lose control of myself: 1 | 0.79 | 0.77 | 0.79 | 0.77 | 0.8 | 0.76 | 0.82 | 0.75 | 0.82 | 0.74 |
| Thinking about how you‚Äôve been feeling more recently, over the last 2 weeks, how often have you b...-Feeling I might lose control of myself: 2 | 0.13 | 0.14 | 0.14 | 0.14 | 0.13 | 0.14 | 0.13 | 0.15 | 0.13 | 0.15 |
| Thinking about how you‚Äôve been feeling more recently, over the last 2 weeks, how often have you b...-Feeling I might lose control of myself: 3 | 0.04 | 0.05 | 0.04 | 0.05 | 0.04 | 0.05 | 0.03 | 0.06 | 0.03 | 0.07 |
| Thinking about how you‚Äôve been feeling more recently, over the last 2 weeks, how often have you b...-Feeling I might lose control of myself: 4 | 0.03 | 0.04 | 0.03 | 0.05 | 0.03 | 0.04 | 0.03 | 0.04 | 0.03 | 0.05 |
| How often have you felt the sentiment stated in each of the sentences below?-I have someone who will listen to me when I need to talk: 1 | 0.01 | 0 | 0 | 0.01 | 0 | 0.01 | 0 | 0.01 | 0 | 0.01 |
| How often have you felt the sentiment stated in each of the sentences below?-I have someone who will listen to me when I need to talk: 2 | 0.03 | 0.06 | 0.03 | 0.05 | 0.03 | 0.05 | 0.03 | 0.05 | 0.03 | 0.05 |
| How often have you felt the sentiment stated in each of the sentences below?-I have someone who will listen to me when I need to talk: 3 | 0.07 | 0.12 | 0.07 | 0.13 | 0.08 | 0.12 | 0.07 | 0.12 | 0.07 | 0.13 |
| How often have you felt the sentiment stated in each of the sentences below?-I have someone who will listen to me when I need to talk: 4 | 0.2 | 0.16 | 0.2 | 0.17 | 0.2 | 0.17 | 0.2 | 0.17 | 0.21 | 0.15 |
| How often have you felt the sentiment stated in each of the sentences below?-I have someone who will listen to me when I need to talk: 5 | 0.69 | 0.65 | 0.69 | 0.65 | 0.68 | 0.66 | 0.69 | 0.66 | 0.68 | 0.66 |
| How often have you felt the sentiment stated in each of the sentences below?-I have someone to confide in or talk to about myself or my problems: 1 | 0.01 | 0.01 | 0 | 0.02 | 0.01 | 0.02 | 0.01 | 0.02 | 0.01 | 0.02 |
| How often have you felt the sentiment stated in each of the sentences below?-I have someone to confide in or talk to about myself or my problems: 2 | 0.03 | 0.05 | 0.03 | 0.05 | 0.03 | 0.05 | 0.03 | 0.04 | 0.03 | 0.04 |
| How often have you felt the sentiment stated in each of the sentences below?-I have someone to confide in or talk to about myself or my problems: 3 | 0.07 | 0.12 | 0.07 | 0.11 | 0.08 | 0.11 | 0.07 | 0.11 | 0.07 | 0.12 |
| How often have you felt the sentiment stated in each of the sentences below?-I have someone to confide in or talk to about myself or my problems: 4 | 0.2 | 0.17 | 0.2 | 0.18 | 0.2 | 0.17 | 0.2 | 0.18 | 0.2 | 0.17 |
| How often have you felt the sentiment stated in each of the sentences below?-I have someone to confide in or talk to about myself or my problems: 5 | 0.69 | 0.64 | 0.69 | 0.64 | 0.69 | 0.65 | 0.69 | 0.65 | 0.69 | 0.65 |
| How often have you felt the sentiment stated in each of the sentences below?-I have someone who makes me feel appreciated: 1 | 0.01 | 0.01 | 0.01 | 0.02 | 0.01 | 0.02 | 0.01 | 0.02 | 0.01 | 0.01 |
| How often have you felt the sentiment stated in each of the sentences below?-I have someone who makes me feel appreciated: 2 | 0.03 | 0.06 | 0.03 | 0.05 | 0.03 | 0.05 | 0.03 | 0.05 | 0.03 | 0.05 |
| How often have you felt the sentiment stated in each of the sentences below?-I have someone who makes me feel appreciated: 3 | 0.08 | 0.12 | 0.08 | 0.13 | 0.08 | 0.13 | 0.08 | 0.12 | 0.08 | 0.13 |
| How often have you felt the sentiment stated in each of the sentences below?-I have someone who makes me feel appreciated: 4 | 0.19 | 0.16 | 0.19 | 0.16 | 0.18 | 0.18 | 0.19 | 0.17 | 0.19 | 0.17 |
| How often have you felt the sentiment stated in each of the sentences below?-I have someone who makes me feel appreciated: 5 | 0.68 | 0.64 | 0.69 | 0.64 | 0.69 | 0.62 | 0.7 | 0.63 | 0.69 | 0.64 |
| How often have you felt the sentiment stated in each of the sentences below?-I have someone to talk with when I have a bad day: 1 | 0.01 | 0.03 | 0.01 | 0.03 | 0.01 | 0.03 | 0 | 0.03 | 0.01 | 0.03 |
| How often have you felt the sentiment stated in each of the sentences below?-I have someone to talk with when I have a bad day: 2 | 0.03 | 0.04 | 0.03 | 0.04 | 0.03 | 0.03 | 0.03 | 0.03 | 0.03 | 0.03 |
| How often have you felt the sentiment stated in each of the sentences below?-I have someone to talk with when I have a bad day: 3 | 0.07 | 0.12 | 0.06 | 0.13 | 0.07 | 0.12 | 0.06 | 0.12 | 0.06 | 0.13 |
| How often have you felt the sentiment stated in each of the sentences below?-I have someone to talk with when I have a bad day: 4 | 0.18 | 0.15 | 0.17 | 0.17 | 0.17 | 0.17 | 0.18 | 0.17 | 0.19 | 0.15 |
| How often have you felt the sentiment stated in each of the sentences below?-I have someone to talk with when I have a bad day: 5 | 0.71 | 0.66 | 0.73 | 0.64 | 0.72 | 0.65 | 0.72 | 0.66 | 0.71 | 0.66 |
| Here are some statements that describe how women may respond to everyday life during pregnancy. ¬†...-That you were unable to control the important things in your life: 1 | 0.3 | 0.35 | 0.3 | 0.33 | 0.3 | 0.33 | 0.3 | 0.33 | 0.31 | 0.31 |
| Here are some statements that describe how women may respond to everyday life during pregnancy. ¬†...-That you were unable to control the important things in your life: 2 | 0.31 | 0.25 | 0.32 | 0.24 | 0.31 | 0.24 | 0.31 | 0.25 | 0.3 | 0.27 |
| Here are some statements that describe how women may respond to everyday life during pregnancy. ¬†...-That you were unable to control the important things in your life: 3 | 0.25 | 0.25 | 0.23 | 0.28 | 0.24 | 0.27 | 0.25 | 0.25 | 0.25 | 0.26 |
| Here are some statements that describe how women may respond to everyday life during pregnancy. ¬†...-That you were unable to control the important things in your life: 4 | 0.08 | 0.1 | 0.09 | 0.09 | 0.09 | 0.09 | 0.07 | 0.11 | 0.08 | 0.11 |
| Here are some statements that describe how women may respond to everyday life during pregnancy. ¬†...-That you were unable to control the important things in your life: 5 | 0.06 | 0.05 | 0.05 | 0.06 | 0.05 | 0.06 | 0.06 | 0.05 | 0.06 | 0.05 |
| Here are some statements that describe how women may respond to everyday life during pregnancy. ¬†...-Confident about your ability to handle your personal problems: 1 | 0.32 | 0.32 | 0.32 | 0.33 | 0.32 | 0.32 | 0.32 | 0.32 | 0.33 | 0.31 |
| Here are some statements that describe how women may respond to everyday life during pregnancy. ¬†...-Confident about your ability to handle your personal problems: 2 | 0.39 | 0.36 | 0.39 | 0.35 | 0.39 | 0.34 | 0.4 | 0.35 | 0.4 | 0.34 |
| Here are some statements that describe how women may respond to everyday life during pregnancy. ¬†...-Confident about your ability to handle your personal problems: 3 | 0.21 | 0.25 | 0.21 | 0.25 | 0.2 | 0.27 | 0.2 | 0.26 | 0.2 | 0.27 |
| Here are some statements that describe how women may respond to everyday life during pregnancy. ¬†...-Confident about your ability to handle your personal problems: 4 | 0.05 | 0.04 | 0.05 | 0.03 | 0.05 | 0.03 | 0.04 | 0.04 | 0.04 | 0.04 |
| Here are some statements that describe how women may respond to everyday life during pregnancy. ¬†...-Confident about your ability to handle your personal problems: 5 | 0.03 | 0.04 | 0.03 | 0.05 | 0.04 | 0.03 | 0.04 | 0.04 | 0.03 | 0.04 |
| Here are some statements that describe how women may respond to everyday life during pregnancy. ¬†...-That things were going your way: 1 | 0.17 | 0.17 | 0.16 | 0.18 | 0.16 | 0.18 | 0.16 | 0.17 | 0.16 | 0.18 |
| Here are some statements that describe how women may respond to everyday life during pregnancy. ¬†...-That things were going your way: 2 | 0.41 | 0.32 | 0.42 | 0.31 | 0.4 | 0.33 | 0.43 | 0.31 | 0.41 | 0.32 |
| Here are some statements that describe how women may respond to everyday life during pregnancy. ¬†...-That things were going your way: 3 | 0.33 | 0.36 | 0.32 | 0.37 | 0.34 | 0.34 | 0.32 | 0.36 | 0.33 | 0.35 |
| Here are some statements that describe how women may respond to everyday life during pregnancy. ¬†...-That things were going your way: 4 | 0.08 | 0.09 | 0.08 | 0.08 | 0.07 | 0.1 | 0.07 | 0.1 | 0.08 | 0.09 |
| Here are some statements that describe how women may respond to everyday life during pregnancy. ¬†...-That things were going your way: 5 | 0.02 | 0.07 | 0.02 | 0.06 | 0.03 | 0.06 | 0.02 | 0.05 | 0.02 | 0.06 |
| Here are some statements that describe how women may respond to everyday life during pregnancy. ¬†...-Difficulties were piling up so high that you could not overcome them: 1 | 0.31 | 0.29 | 0.3 | 0.31 | 0.32 | 0.28 | 0.31 | 0.29 | 0.31 | 0.29 |
| Here are some statements that describe how women may respond to everyday life during pregnancy. ¬†...-Difficulties were piling up so high that you could not overcome them: 2 | 0.37 | 0.34 | 0.37 | 0.33 | 0.36 | 0.34 | 0.38 | 0.33 | 0.37 | 0.33 |
| Here are some statements that describe how women may respond to everyday life during pregnancy. ¬†...-Difficulties were piling up so high that you could not overcome them: 3 | 0.25 | 0.25 | 0.26 | 0.24 | 0.25 | 0.25 | 0.25 | 0.26 | 0.25 | 0.26 |
| Here are some statements that describe how women may respond to everyday life during pregnancy. ¬†...-Difficulties were piling up so high that you could not overcome them: 4 | 0.05 | 0.1 | 0.05 | 0.09 | 0.06 | 0.09 | 0.05 | 0.09 | 0.06 | 0.08 |
| Here are some statements that describe how women may respond to everyday life during pregnancy. ¬†...-Difficulties were piling up so high that you could not overcome them: 5 | 0.02 | 0.03 | 0.01 | 0.04 | 0.01 | 0.04 | 0.01 | 0.03 | 0.01 | 0.03 |
| Congratulations! How far along are you in your pregnancy?: 4 | 0.14 | 0.19 | 0.16 | 0.17 | 0.15 | 0.17 | 0.15 | 0.17 | 0.15 | 0.17 |
| Congratulations! How far along are you in your pregnancy?: 5 | 0.21 | 0.23 | 0.21 | 0.23 | 0.2 | 0.25 | 0.21 | 0.23 | 0.21 | 0.23 |
| Congratulations! How far along are you in your pregnancy?: 6 | 0.19 | 0.16 | 0.19 | 0.18 | 0.19 | 0.16 | 0.19 | 0.18 | 0.19 | 0.17 |
| Congratulations! How far along are you in your pregnancy?: 7 | 0.11 | 0.14 | 0.11 | 0.13 | 0.11 | 0.13 | 0.11 | 0.13 | 0.1 | 0.14 |
| Congratulations! How far along are you in your pregnancy?: 8 | 0.14 | 0.12 | 0.13 | 0.13 | 0.12 | 0.14 | 0.13 | 0.12 | 0.13 | 0.13 |
| Congratulations! How far along are you in your pregnancy?: 9 | 0.11 | 0.09 | 0.11 | 0.1 | 0.12 | 0.08 | 0.12 | 0.09 | 0.11 | 0.09 |
| Congratulations! How far along are you in your pregnancy?: 10 | 0.09 | 0.07 | 0.1 | 0.07 | 0.1 | 0.07 | 0.1 | 0.07 | 0.1 | 0.06 |
| device: desktop | 0.33 | 0.21 | 0.34 | 0.2 | 0.33 | 0.2 | 0.35 | 0.21 | 0.34 | 0.2 |
| device: mobile | 0.67 | 0.79 | 0.66 | 0.8 | 0.67 | 0.8 | 0.65 | 0.79 | 0.66 | 0.8 |
| For each of the statements below please pick the response that best describes your present feelin...-I feel calm: 1 | 0.32 | 0.33 | 0.32 | 0.33 | 0.32 | 0.33 | 0.32 | 0.33 | 0.32 | 0.34 |
| For each of the statements below please pick the response that best describes your present feelin...-I feel calm: 2 | 0.38 | 0.31 | 0.38 | 0.32 | 0.37 | 0.32 | 0.38 | 0.32 | 0.37 | 0.32 |
| For each of the statements below please pick the response that best describes your present feelin...-I feel calm: 3 | 0.27 | 0.33 | 0.27 | 0.31 | 0.28 | 0.31 | 0.27 | 0.31 | 0.28 | 0.31 |
| For each of the statements below please pick the response that best describes your present feelin...-I feel calm: 4 | 0.03 | 0.03 | 0.03 | 0.04 | 0.03 | 0.04 | 0.03 | 0.04 | 0.03 | 0.03 |
| For each of the statements below please pick the response that best describes your present feelin...-I am tense: 1 | 0.42 | 0.43 | 0.41 | 0.46 | 0.42 | 0.44 | 0.43 | 0.42 | 0.42 | 0.44 |
| For each of the statements below please pick the response that best describes your present feelin...-I am tense: 2 | 0.41 | 0.38 | 0.41 | 0.38 | 0.41 | 0.39 | 0.41 | 0.39 | 0.42 | 0.38 |
| For each of the statements below please pick the response that best describes your present feelin...-I am tense: 3 | 0.13 | 0.15 | 0.14 | 0.13 | 0.13 | 0.14 | 0.12 | 0.15 | 0.13 | 0.14 |
| For each of the statements below please pick the response that best describes your present feelin...-I am tense: 4 | 0.04 | 0.04 | 0.04 | 0.03 | 0.04 | 0.04 | 0.04 | 0.04 | 0.03 | 0.04 |
| For each of the statements below please pick the response that best describes your present feelin...-I feel upset: 1 | 0.72 | 0.6 | 0.71 | 0.61 | 0.73 | 0.57 | 0.74 | 0.59 | 0.74 | 0.58 |
| For each of the statements below please pick the response that best describes your present feelin...-I feel upset: 2 | 0.2 | 0.28 | 0.2 | 0.28 | 0.19 | 0.3 | 0.19 | 0.28 | 0.2 | 0.28 |
| For each of the statements below please pick the response that best describes your present feelin...-I feel upset: 3 | 0.07 | 0.09 | 0.08 | 0.08 | 0.06 | 0.1 | 0.05 | 0.11 | 0.05 | 0.11 |
| For each of the statements below please pick the response that best describes your present feelin...-I feel upset: 4 | 0.01 | 0.03 | 0.01 | 0.03 | 0.02 | 0.03 | 0.01 | 0.03 | 0.01 | 0.03 |
| For each of the statements below please pick the response that best describes your present feelin...-I am relaxed: 1 | 0.18 | 0.2 | 0.17 | 0.2 | 0.17 | 0.22 | 0.17 | 0.2 | 0.17 | 0.21 |
| For each of the statements below please pick the response that best describes your present feelin...-I am relaxed: 2 | 0.39 | 0.34 | 0.38 | 0.35 | 0.38 | 0.34 | 0.39 | 0.35 | 0.38 | 0.35 |
| For each of the statements below please pick the response that best describes your present feelin...-I am relaxed: 3 | 0.36 | 0.38 | 0.37 | 0.36 | 0.38 | 0.34 | 0.38 | 0.35 | 0.37 | 0.36 |
| For each of the statements below please pick the response that best describes your present feelin...-I am relaxed: 4 | 0.08 | 0.08 | 0.07 | 0.08 | 0.07 | 0.1 | 0.06 | 0.1 | 0.07 | 0.09 |
| For each of the statements below please pick the response that best describes your present feelin...-I feel content: 1 | 0.34 | 0.32 | 0.34 | 0.32 | 0.35 | 0.31 | 0.33 | 0.33 | 0.34 | 0.33 |
| For each of the statements below please pick the response that best describes your present feelin...-I feel content: 2 | 0.38 | 0.33 | 0.37 | 0.35 | 0.37 | 0.35 | 0.39 | 0.32 | 0.38 | 0.33 |
| For each of the statements below please pick the response that best describes your present feelin...-I feel content: 3 | 0.23 | 0.3 | 0.24 | 0.27 | 0.23 | 0.29 | 0.23 | 0.28 | 0.23 | 0.28 |
| For each of the statements below please pick the response that best describes your present feelin...-I feel content: 4 | 0.05 | 0.06 | 0.05 | 0.06 | 0.05 | 0.06 | 0.05 | 0.06 | 0.05 | 0.06 |
| For each of the statements below please pick the response that best describes your present feelin...-I am worried: 1 | 0.18 | 0.23 | 0.19 | 0.22 | 0.2 | 0.2 | 0.2 | 0.2 | 0.2 | 0.2 |
| For each of the statements below please pick the response that best describes your present feelin...-I am worried: 2 | 0.49 | 0.44 | 0.5 | 0.43 | 0.48 | 0.47 | 0.49 | 0.45 | 0.49 | 0.45 |
| For each of the statements below please pick the response that best describes your present feelin...-I am worried: 3 | 0.23 | 0.19 | 0.21 | 0.22 | 0.22 | 0.2 | 0.21 | 0.22 | 0.21 | 0.22 |
| For each of the statements below please pick the response that best describes your present feelin...-I am worried: 4 | 0.1 | 0.13 | 0.11 | 0.12 | 0.1 | 0.13 | 0.1 | 0.13 | 0.1 | 0.13 |
| How old are you?: 2 | 0.2 | 0.27 | 0.2 | 0.28 | 0.19 | 0.3 | 0.19 | 0.28 | 0.19 | 0.29 |
| How old are you?: 3 | 0.29 | 0.32 | 0.32 | 0.26 | 0.32 | 0.25 | 0.3 | 0.29 | 0.31 | 0.27 |
| How old are you?: 4 | 0.31 | 0.26 | 0.29 | 0.29 | 0.3 | 0.29 | 0.3 | 0.28 | 0.3 | 0.29 |
| How old are you?: 5 | 0.17 | 0.11 | 0.16 | 0.12 | 0.17 | 0.11 | 0.17 | 0.12 | 0.17 | 0.11 |
| How old are you?: 6 | 0.03 | 0.05 | 0.03 | 0.05 | 0.03 | 0.05 | 0.03 | 0.04 | 0.03 | 0.04 |
| To begin, which statement best describes your reaction when you first found out you were pregnant?: 1 | 0.34 | 0.3 | 0.34 | 0.3 | 0.34 | 0.31 | 0.34 | 0.31 | 0.34 | 0.3 |
| To begin, which statement best describes your reaction when you first found out you were pregnant?: 2 | 0.28 | 0.24 | 0.26 | 0.27 | 0.28 | 0.24 | 0.27 | 0.26 | 0.26 | 0.27 |
| To begin, which statement best describes your reaction when you first found out you were pregnant?: 3 | 0.03 | 0.02 | 0.03 | 0.02 | 0.03 | 0.02 | 0.03 | 0.03 | 0.03 | 0.02 |
| To begin, which statement best describes your reaction when you first found out you were pregnant?: 4 | 0 | 0.01 | 0 | 0.01 | 0 | 0.01 | 0 | 0.01 | 0 | 0.01 |
| To begin, which statement best describes your reaction when you first found out you were pregnant?: 5 | 0.01 | 0.01 | 0.01 | 0.01 | 0.01 | 0.01 | 0.01 | 0.02 | 0.01 | 0.02 |
| To begin, which statement best describes your reaction when you first found out you were pregnant?: 6 | 0.33 | 0.42 | 0.34 | 0.39 | 0.34 | 0.41 | 0.36 | 0.37 | 0.35 | 0.38 |
| How have you been feeling so far in this pregnancy?: 1 | 0.17 | 0.18 | 0.16 | 0.19 | 0.16 | 0.2 | 0.15 | 0.2 | 0.15 | 0.2 |
| How have you been feeling so far in this pregnancy?: 2 | 0.63 | 0.62 | 0.65 | 0.59 | 0.64 | 0.59 | 0.65 | 0.6 | 0.65 | 0.59 |
| How have you been feeling so far in this pregnancy?: 3 | 0.16 | 0.15 | 0.16 | 0.17 | 0.16 | 0.16 | 0.17 | 0.15 | 0.17 | 0.15 |
| How have you been feeling so far in this pregnancy?: 4 | 0.03 | 0.05 | 0.03 | 0.05 | 0.04 | 0.05 | 0.03 | 0.05 | 0.03 | 0.06 |
| Have you experienced any of the following during this pregnancy? (Please select all that apply.)-Nausea | 0.65 | 0.66 | 0.65 | 0.65 | 0.65 | 0.65 | 0.68 | 0.62 | 0.67 | 0.63 |
| Have you experienced any of the following during this pregnancy? (Please select all that apply.)-Anxiety | 0.21 | 0.21 | 0.2 | 0.22 | 0.2 | 0.22 | 0.2 | 0.22 | 0.21 | 0.21 |
| Have you experienced any of the following during this pregnancy? (Please select all that apply.)-Depression | 0.09 | 0.11 | 0.09 | 0.12 | 0.09 | 0.12 | 0.08 | 0.13 | 0.09 | 0.12 |
| Have you experienced any of the following during this pregnancy? (Please select all that apply.)-High blood pressure | 0.03 | 0.03 | 0.02 | 0.03 | 0.03 | 0.03 | 0.03 | 0.03 | 0.03 | 0.03 |
| Have you experienced any of the following during this pregnancy? (Please select all that apply.)-Other, please specify: | 0.11 | 0.1 | 0.11 | 0.1 | 0.11 | 0.1 | 0.12 | 0.1 | 0.12 | 0.1 |
| Have you experienced any of the following during this pregnancy? (Please select all that apply.)-None of these | 0.03 | 0.02 | 0.02 | 0.02 | 0.03 | 0.01 | 0.03 | 0.02 | 0.03 | 0.02 |
| Have you experienced any of the following during this pregnancy? (Please select all that apply.)-Difficulty eating (e.g., motivation to eat healthy, appetite) | 0.43 | 0.35 | 0.43 | 0.36 | 0.42 | 0.37 | 0.43 | 0.37 | 0.43 | 0.36 |
| Have you experienced any of the following during this pregnancy? (Please select all that apply.)-Fatigue or lack of energy | 0.87 | 0.8 | 0.87 | 0.8 | 0.86 | 0.81 | 0.86 | 0.82 | 0.86 | 0.81 |
| Have you experienced any of the following during this pregnancy? (Please select all that apply.)-Back pain | 0.38 | 0.39 | 0.37 | 0.4 | 0.37 | 0.41 | 0.35 | 0.42 | 0.36 | 0.42 |
| Have you experienced any of the following during this pregnancy? (Please select all that apply.)-Headaches or migraines | 0.37 | 0.4 | 0.38 | 0.39 | 0.38 | 0.38 | 0.38 | 0.38 | 0.37 | 0.4 |
| Have you experienced any of the following during this pregnancy? (Please select all that apply.)-Insomnia or trouble sleeping | 0.37 | 0.34 | 0.38 | 0.32 | 0.37 | 0.33 | 0.37 | 0.35 | 0.36 | 0.35 |
| Have you experienced any of the following during this pregnancy? (Please select all that apply.)-Mood swings | 0.48 | 0.5 | 0.48 | 0.49 | 0.48 | 0.5 | 0.47 | 0.51 | 0.48 | 0.5 |
| Have you ever been pregnant before?: No | 0.35 | 0.32 | 0.34 | 0.33 | 0.34 | 0.34 | 0.32 | 0.36 | 0.32 | 0.36 |
| Have you ever been pregnant before?: Yes | 0.65 | 0.68 | 0.66 | 0.67 | 0.66 | 0.66 | 0.68 | 0.64 | 0.68 | 0.64 |
| Did you experience any of the following in previous pregnancies?¬† (Please select all that apply.)-Nausea | 0.46 | 0.49 | 0.47 | 0.48 | 0.48 | 0.46 | 0.49 | 0.45 | 0.49 | 0.45 |
| Did you experience any of the following in previous pregnancies?¬† (Please select all that apply.)-Depression | 0.09 | 0.12 | 0.1 | 0.11 | 0.1 | 0.11 | 0.1 | 0.11 | 0.1 | 0.11 |
| Did you experience any of the following in previous pregnancies?¬† (Please select all that apply.)-Gestational diabetes | 0.04 | 0.05 | 0.04 | 0.04 | 0.04 | 0.04 | 0.04 | 0.04 | 0.04 | 0.03 |
| Did you experience any of the following in previous pregnancies?¬† (Please select all that apply.)-High blood pressure | 0.06 | 0.06 | 0.06 | 0.06 | 0.06 | 0.06 | 0.07 | 0.05 | 0.07 | 0.05 |
| Did you experience any of the following in previous pregnancies?¬† (Please select all that apply.)-Preeclampsia | 0.05 | 0.05 | 0.04 | 0.05 | 0.05 | 0.05 | 0.05 | 0.04 | 0.05 | 0.05 |
| Did you experience any of the following in previous pregnancies?¬† (Please select all that apply.)-Miscarriage | 0.17 | 0.23 | 0.19 | 0.2 | 0.18 | 0.22 | 0.19 | 0.2 | 0.2 | 0.18 |
| Did you experience any of the following in previous pregnancies?¬† (Please select all that apply.)-Pre-term birth | 0.06 | 0.07 | 0.05 | 0.08 | 0.06 | 0.07 | 0.06 | 0.08 | 0.06 | 0.07 |
| Did you experience any of the following in previous pregnancies?¬† (Please select all that apply.)-Stillbirth | 0.01 | 0.02 | 0.01 | 0.02 | 0.01 | 0.03 | 0.01 | 0.02 | 0.01 | 0.02 |
| Did you experience any of the following in previous pregnancies?¬† (Please select all that apply.)-Delivery complications | 0.09 | 0.09 | 0.09 | 0.08 | 0.1 | 0.08 | 0.1 | 0.07 | 0.1 | 0.07 |
| Did you experience any of the following in previous pregnancies?¬† (Please select all that apply.)-Baby placed in NICU | 0.08 | 0.09 | 0.08 | 0.09 | 0.08 | 0.08 | 0.08 | 0.08 | 0.09 | 0.08 |
| Did you experience any of the following in previous pregnancies?¬† (Please select all that apply.)-Other, please specify: | 0.04 | 0.02 | 0.04 | 0.03 | 0.03 | 0.03 | 0.04 | 0.03 | 0.03 | 0.03 |
| Did you experience any of the following in previous pregnancies?¬† (Please select all that apply.)-Difficulty eating (e.g., motivation to eat healthy, appetite) | 0.23 | 0.21 | 0.24 | 0.21 | 0.24 | 0.21 | 0.25 | 0.2 | 0.25 | 0.2 |
| Did you experience any of the following in previous pregnancies?¬† (Please select all that apply.)-None of these | 0.02 | 0.04 | 0.02 | 0.04 | 0.02 | 0.03 | 0.02 | 0.03 | 0.02 | 0.03 |
| Did you experience any of the following in previous pregnancies?¬† (Please select all that apply.)-Difficulty managing your weight | 0.18 | 0.17 | 0.19 | 0.16 | 0.19 | 0.16 | 0.19 | 0.16 | 0.19 | 0.15 |
| Did you experience any of the following in previous pregnancies?¬† (Please select all that apply.)-Fatigue or lack of energy | 0.48 | 0.43 | 0.49 | 0.42 | 0.49 | 0.42 | 0.49 | 0.43 | 0.49 | 0.42 |
| Did you experience any of the following in previous pregnancies?¬† (Please select all that apply.)-Back pain | 0.3 | 0.29 | 0.31 | 0.28 | 0.32 | 0.27 | 0.32 | 0.28 | 0.32 | 0.27 |
| Did you experience any of the following in previous pregnancies?¬† (Please select all that apply.)-Headaches or migraines | 0.2 | 0.17 | 0.22 | 0.15 | 0.21 | 0.15 | 0.21 | 0.17 | 0.2 | 0.18 |
| Did you experience any of the following in previous pregnancies?¬† (Please select all that apply.)-Insomnia or trouble sleeping | 0.26 | 0.2 | 0.26 | 0.2 | 0.26 | 0.19 | 0.26 | 0.21 | 0.26 | 0.2 |
| Did you experience any of the following in previous pregnancies?¬† (Please select all that apply.)-Mood swings | 0.27 | 0.36 | 0.28 | 0.33 | 0.29 | 0.32 | 0.29 | 0.32 | 0.29 | 0.32 |
| Did you experience any of the following in previous pregnancies?¬† (Please select all that apply.)-Anxiety | 0.11 | 0.13 | 0.13 | 0.11 | 0.13 | 0.11 | 0.13 | 0.11 | 0.13 | 0.11 |
| Have you seen an obstetrician or other health care provider for prenatal care for this pregnancy?: No, but I plan to make a future appointment | 0.14 | 0.2 | 0.14 | 0.19 | 0.14 | 0.2 | 0.14 | 0.18 | 0.14 | 0.19 |
| Have you seen an obstetrician or other health care provider for prenatal care for this pregnancy?: No, I have no plans to seek prenatal care from a health care provider | 0 | 0.02 | 0 | 0.02 | 0 | 0.02 | NA | NA | NA | NA |
| Have you seen an obstetrician or other health care provider for prenatal care for this pregnancy?: No, it‚Äôs too early in my pregnancy, but I do have a future appointment | 0.42 | 0.35 | 0.43 | 0.35 | 0.42 | 0.36 | 0.41 | 0.39 | 0.41 | 0.38 |
| Have you seen an obstetrician or other health care provider for prenatal care for this pregnancy?: Yes, I have had a prenatal checkup, but don‚Äôt plan for regular appointments | 0.01 | 0.01 | 0.01 | 0.01 | 0.01 | 0.01 | 0.01 | 0.01 | 0.01 | 0.01 |
| Have you seen an obstetrician or other health care provider for prenatal care for this pregnancy?: Yes, I have regularly scheduled appointments for prenatal care | 0.43 | 0.42 | 0.42 | 0.44 | 0.43 | 0.41 | 0.43 | 0.41 | 0.44 | 0.4 |
| How healthy do you eat now that you‚Äôre pregnant?: 1 | 0.05 | 0.03 | 0.04 | 0.04 | 0.05 | 0.03 | 0.05 | 0.03 | 0.05 | 0.03 |
| How healthy do you eat now that you‚Äôre pregnant?: 2 | 0.25 | 0.17 | 0.24 | 0.2 | 0.24 | 0.2 | 0.24 | 0.21 | 0.24 | 0.2 |
| How healthy do you eat now that you‚Äôre pregnant?: 3 | 0.6 | 0.58 | 0.61 | 0.56 | 0.62 | 0.54 | 0.62 | 0.56 | 0.61 | 0.56 |
| How healthy do you eat now that you‚Äôre pregnant?: 4 | 0.1 | 0.2 | 0.11 | 0.19 | 0.1 | 0.21 | 0.09 | 0.19 | 0.09 | 0.21 |
| How healthy do you eat now that you‚Äôre pregnant?: 5 | 0 | 0.01 | 0 | 0.01 | 0 | 0.01 | 0 | 0.01 | 0 | 0.01 |
| Which of these benefits of pregnancy are you most looking forward to? (Please select all that app...-Becoming more health-conscious | 0.51 | 0.47 | 0.52 | 0.45 | 0.51 | 0.47 | 0.54 | 0.44 | 0.52 | 0.46 |
| Which of these benefits of pregnancy are you most looking forward to? (Please select all that app...-Others holding doors open or giving up seats for me | 0.08 | 0.08 | 0.08 | 0.09 | 0.09 | 0.08 | 0.08 | 0.08 | 0.09 | 0.08 |
| Which of these benefits of pregnancy are you most looking forward to? (Please select all that app...-Extra lush hair and strong nails | 0.32 | 0.35 | 0.31 | 0.37 | 0.32 | 0.35 | 0.3 | 0.37 | 0.32 | 0.36 |
| Which of these benefits of pregnancy are you most looking forward to? (Please select all that app...-No need for birth control | 0.21 | 0.17 | 0.22 | 0.16 | 0.22 | 0.17 | 0.21 | 0.19 | 0.22 | 0.16 |
| Which of these benefits of pregnancy are you most looking forward to? (Please select all that app...-Eating ‚Äúwhat I want‚Äù | 0.15 | 0.15 | 0.13 | 0.18 | 0.14 | 0.17 | 0.12 | 0.18 | 0.13 | 0.18 |
| Which of these benefits of pregnancy are you most looking forward to? (Please select all that app...-Extra cleavage | 0.16 | 0.15 | 0.16 | 0.15 | 0.15 | 0.16 | 0.15 | 0.17 | 0.15 | 0.16 |
| Which of these benefits of pregnancy are you most looking forward to? (Please select all that app...-Buying new clothes/shoes | 0.22 | 0.21 | 0.22 | 0.22 | 0.21 | 0.23 | 0.2 | 0.24 | 0.2 | 0.24 |
| Which of these benefits of pregnancy are you most looking forward to? (Please select all that app...-None of these | 0.24 | 0.26 | 0.24 | 0.26 | 0.25 | 0.25 | 0.23 | 0.26 | 0.24 | 0.26 |
| How bad is ‚Äúmorning sickness‚Äù for you during this pregnancy?: 1 | 0.1 | 0.13 | 0.1 | 0.12 | 0.09 | 0.14 | 0.1 | 0.12 | 0.1 | 0.13 |
| How bad is ‚Äúmorning sickness‚Äù for you during this pregnancy?: 2 | 0.17 | 0.13 | 0.15 | 0.17 | 0.16 | 0.15 | 0.16 | 0.15 | 0.17 | 0.14 |
| How bad is ‚Äúmorning sickness‚Äù for you during this pregnancy?: 3 | 0.4 | 0.35 | 0.42 | 0.32 | 0.41 | 0.32 | 0.43 | 0.32 | 0.41 | 0.33 |
| How bad is ‚Äúmorning sickness‚Äù for you during this pregnancy?: 4 | 0.01 | 0.01 | 0.01 | 0.01 | 0.02 | 0.01 | 0.01 | 0.01 | 0.02 | 0.01 |
| How bad is ‚Äúmorning sickness‚Äù for you during this pregnancy?: 5 | 0.32 | 0.38 | 0.32 | 0.38 | 0.32 | 0.4 | 0.3 | 0.4 | 0.31 | 0.4 |
| How would you describe your general overall health prior to becoming pregnant?¬†: 1 | 0.19 | 0.11 | 0.17 | 0.15 | 0.17 | 0.14 | 0.17 | 0.14 | 0.18 | 0.14 |
| How would you describe your general overall health prior to becoming pregnant?¬†: 2 | 0.42 | 0.35 | 0.43 | 0.34 | 0.41 | 0.37 | 0.41 | 0.38 | 0.41 | 0.36 |
| How would you describe your general overall health prior to becoming pregnant?¬†: 3 | 0.29 | 0.39 | 0.31 | 0.35 | 0.31 | 0.36 | 0.32 | 0.34 | 0.31 | 0.36 |
| How would you describe your general overall health prior to becoming pregnant?¬†: 4 | 0.09 | 0.11 | 0.08 | 0.12 | 0.09 | 0.1 | 0.09 | 0.11 | 0.09 | 0.1 |
| How would you describe your general overall health prior to becoming pregnant?¬†: 5 | 0.02 | 0.04 | 0.02 | 0.04 | 0.02 | 0.04 | 0.01 | 0.03 | 0.01 | 0.04 |
| How would you describe your weight before you became pregnant?: 1 | 0 | 0 | 0 | 0.01 | 0 | 0 | 0 | 0.01 | 0 | 0.01 |
| How would you describe your weight before you became pregnant?: 2 | 0.01 | 0.02 | 0.01 | 0.02 | 0.01 | 0.02 | 0.01 | 0.02 | 0.01 | 0.02 |
| How would you describe your weight before you became pregnant?: 3 | 0.05 | 0.05 | 0.05 | 0.05 | 0.06 | 0.04 | 0.06 | 0.04 | 0.05 | 0.05 |
| How would you describe your weight before you became pregnant?: 4 | 0.41 | 0.36 | 0.4 | 0.37 | 0.39 | 0.39 | 0.39 | 0.4 | 0.39 | 0.39 |
| How would you describe your weight before you became pregnant?: 5 | 0.26 | 0.33 | 0.27 | 0.3 | 0.28 | 0.3 | 0.28 | 0.3 | 0.28 | 0.29 |
| How would you describe your weight before you became pregnant?: 6 | 0.21 | 0.19 | 0.21 | 0.19 | 0.21 | 0.19 | 0.21 | 0.2 | 0.21 | 0.2 |
| How would you describe your weight before you became pregnant?: 7 | 0.05 | 0.06 | 0.05 | 0.05 | 0.05 | 0.05 | 0.05 | 0.04 | 0.05 | 0.04 |
| How frequently did you do the following prior to becoming pregnant?-Drink alcohol: 1 | 0.02 | 0.03 | 0.03 | 0.02 | 0.03 | 0.02 | 0.02 | 0.02 | 0.02 | 0.02 |
| How frequently did you do the following prior to becoming pregnant?-Drink alcohol: 2 | 0.12 | 0.13 | 0.12 | 0.12 | 0.13 | 0.11 | 0.13 | 0.12 | 0.13 | 0.11 |
| How frequently did you do the following prior to becoming pregnant?-Drink alcohol: 3 | 0.15 | 0.13 | 0.15 | 0.13 | 0.14 | 0.15 | 0.14 | 0.15 | 0.14 | 0.15 |
| How frequently did you do the following prior to becoming pregnant?-Drink alcohol: 4 | 0.3 | 0.24 | 0.3 | 0.25 | 0.31 | 0.22 | 0.3 | 0.25 | 0.31 | 0.22 |
| How frequently did you do the following prior to becoming pregnant?-Drink alcohol: 5 | 0.41 | 0.47 | 0.4 | 0.48 | 0.39 | 0.5 | 0.41 | 0.46 | 0.39 | 0.49 |
| How frequently did you do the following prior to becoming pregnant?-Read a book: 1 | 0.14 | 0.12 | 0.14 | 0.11 | 0.14 | 0.12 | 0.14 | 0.12 | 0.14 | 0.11 |
| How frequently did you do the following prior to becoming pregnant?-Read a book: 2 | 0.23 | 0.16 | 0.23 | 0.17 | 0.24 | 0.16 | 0.24 | 0.17 | 0.23 | 0.17 |
| How frequently did you do the following prior to becoming pregnant?-Read a book: 3 | 0.16 | 0.14 | 0.16 | 0.14 | 0.16 | 0.14 | 0.16 | 0.14 | 0.17 | 0.13 |
| How frequently did you do the following prior to becoming pregnant?-Read a book: 4 | 0.33 | 0.35 | 0.34 | 0.33 | 0.33 | 0.35 | 0.34 | 0.34 | 0.33 | 0.35 |
| How frequently did you do the following prior to becoming pregnant?-Read a book: 5 | 0.14 | 0.24 | 0.13 | 0.25 | 0.14 | 0.24 | 0.13 | 0.23 | 0.13 | 0.24 |
| How frequently did you do the following prior to becoming pregnant?-Participate in an online discussion group: 1 | 0.05 | 0.05 | 0.04 | 0.06 | 0.05 | 0.06 | 0.05 | 0.05 | 0.05 | 0.05 |
| How frequently did you do the following prior to becoming pregnant?-Participate in an online discussion group: 2 | 0.06 | 0.05 | 0.07 | 0.04 | 0.06 | 0.04 | 0.07 | 0.04 | 0.07 | 0.04 |
| How frequently did you do the following prior to becoming pregnant?-Participate in an online discussion group: 3 | 0.06 | 0.07 | 0.06 | 0.06 | 0.06 | 0.05 | 0.06 | 0.06 | 0.06 | 0.06 |
| How frequently did you do the following prior to becoming pregnant?-Participate in an online discussion group: 4 | 0.17 | 0.14 | 0.19 | 0.12 | 0.17 | 0.13 | 0.18 | 0.14 | 0.18 | 0.13 |
| How frequently did you do the following prior to becoming pregnant?-Participate in an online discussion group: 5 | 0.66 | 0.7 | 0.64 | 0.73 | 0.65 | 0.72 | 0.65 | 0.71 | 0.65 | 0.72 |
| How frequently did you do the following prior to becoming pregnant?-Meet with friends: 1 | 0.04 | 0.08 | 0.04 | 0.08 | 0.04 | 0.09 | 0.04 | 0.08 | 0.04 | 0.08 |
| How frequently did you do the following prior to becoming pregnant?-Meet with friends: 2 | 0.22 | 0.23 | 0.23 | 0.22 | 0.24 | 0.19 | 0.24 | 0.21 | 0.24 | 0.2 |
| How frequently did you do the following prior to becoming pregnant?-Meet with friends: 3 | 0.31 | 0.27 | 0.31 | 0.27 | 0.3 | 0.28 | 0.3 | 0.28 | 0.3 | 0.28 |
| How frequently did you do the following prior to becoming pregnant?-Meet with friends: 4 | 0.35 | 0.31 | 0.35 | 0.32 | 0.35 | 0.32 | 0.36 | 0.32 | 0.35 | 0.33 |
| How frequently did you do the following prior to becoming pregnant?-Meet with friends: 5 | 0.07 | 0.12 | 0.07 | 0.12 | 0.07 | 0.12 | 0.06 | 0.12 | 0.07 | 0.12 |
| How frequently did you do the following prior to becoming pregnant?-Eat junk food: 1 | 0.09 | 0.16 | 0.09 | 0.16 | 0.09 | 0.16 | 0.09 | 0.15 | 0.1 | 0.15 |
| How frequently did you do the following prior to becoming pregnant?-Eat junk food: 2 | 0.39 | 0.42 | 0.38 | 0.44 | 0.39 | 0.42 | 0.39 | 0.42 | 0.38 | 0.43 |
| How frequently did you do the following prior to becoming pregnant?-Eat junk food: 3 | 0.23 | 0.18 | 0.24 | 0.17 | 0.23 | 0.18 | 0.24 | 0.18 | 0.23 | 0.18 |
| How frequently did you do the following prior to becoming pregnant?-Eat junk food: 4 | 0.27 | 0.2 | 0.27 | 0.2 | 0.27 | 0.19 | 0.26 | 0.22 | 0.27 | 0.19 |
| How frequently did you do the following prior to becoming pregnant?-Eat junk food: 5 | 0.03 | 0.04 | 0.03 | 0.04 | 0.02 | 0.05 | 0.02 | 0.04 | 0.02 | 0.04 |
| How frequently did you do the following prior to becoming pregnant?-Go for walks: 1 | 0.19 | 0.2 | 0.19 | 0.2 | 0.18 | 0.21 | 0.18 | 0.2 | 0.19 | 0.19 |
| How frequently did you do the following prior to becoming pregnant?-Go for walks: 2 | 0.38 | 0.29 | 0.38 | 0.3 | 0.38 | 0.29 | 0.39 | 0.3 | 0.38 | 0.3 |
| How frequently did you do the following prior to becoming pregnant?-Go for walks: 3 | 0.16 | 0.17 | 0.16 | 0.17 | 0.17 | 0.16 | 0.16 | 0.17 | 0.16 | 0.17 |
| How frequently did you do the following prior to becoming pregnant?-Go for walks: 4 | 0.22 | 0.25 | 0.22 | 0.25 | 0.22 | 0.25 | 0.22 | 0.25 | 0.22 | 0.25 |
| How frequently did you do the following prior to becoming pregnant?-Go for walks: 5 | 0.05 | 0.09 | 0.05 | 0.08 | 0.05 | 0.09 | 0.04 | 0.09 | 0.05 | 0.09 |
| How frequently did you do the following prior to becoming pregnant?-Practice meditation or breathing: 1 | 0.05 | 0.06 | 0.05 | 0.07 | 0.05 | 0.06 | 0.04 | 0.06 | 0.05 | 0.06 |
| How frequently did you do the following prior to becoming pregnant?-Practice meditation or breathing: 2 | 0.1 | 0.1 | 0.11 | 0.08 | 0.11 | 0.08 | 0.11 | 0.08 | 0.11 | 0.08 |
| How frequently did you do the following prior to becoming pregnant?-Practice meditation or breathing: 3 | 0.08 | 0.08 | 0.08 | 0.07 | 0.07 | 0.08 | 0.07 | 0.08 | 0.08 | 0.07 |
| How frequently did you do the following prior to becoming pregnant?-Practice meditation or breathing: 4 | 0.23 | 0.18 | 0.23 | 0.2 | 0.24 | 0.17 | 0.24 | 0.19 | 0.24 | 0.18 |
| How frequently did you do the following prior to becoming pregnant?-Practice meditation or breathing: 5 | 0.54 | 0.58 | 0.54 | 0.58 | 0.53 | 0.61 | 0.53 | 0.59 | 0.53 | 0.6 |
| How frequently did you do the following prior to becoming pregnant?-Smoke cigarettes: 1 | 0.08 | 0.12 | 0.08 | 0.11 | 0.08 | 0.13 | 0.08 | 0.11 | 0.08 | 0.12 |
| How frequently did you do the following prior to becoming pregnant?-Smoke cigarettes: 2 | 0.03 | 0.03 | 0.03 | 0.03 | 0.03 | 0.03 | 0.02 | 0.04 | 0.02 | 0.04 |
| How frequently did you do the following prior to becoming pregnant?-Smoke cigarettes: 3 | 0 | 0.01 | 0 | 0.01 | 0.01 | 0 | 0.01 | 0 | 0.01 | 0 |
| How frequently did you do the following prior to becoming pregnant?-Smoke cigarettes: 4 | 0.04 | 0.05 | 0.05 | 0.04 | 0.04 | 0.05 | 0.04 | 0.05 | 0.05 | 0.04 |
| How frequently did you do the following prior to becoming pregnant?-Smoke cigarettes: 5 | 0.84 | 0.8 | 0.84 | 0.81 | 0.85 | 0.78 | 0.85 | 0.8 | 0.85 | 0.8 |
| How frequently did you do the following prior to becoming pregnant?-Exercise: 1 | 0.12 | 0.1 | 0.12 | 0.11 | 0.1 | 0.14 | 0.11 | 0.12 | 0.11 | 0.12 |
| How frequently did you do the following prior to becoming pregnant?-Exercise: 2 | 0.41 | 0.33 | 0.4 | 0.35 | 0.41 | 0.32 | 0.42 | 0.34 | 0.41 | 0.34 |
| How frequently did you do the following prior to becoming pregnant?-Exercise: 3 | 0.16 | 0.15 | 0.17 | 0.14 | 0.16 | 0.16 | 0.17 | 0.14 | 0.16 | 0.15 |
| How frequently did you do the following prior to becoming pregnant?-Exercise: 4 | 0.23 | 0.3 | 0.24 | 0.28 | 0.25 | 0.27 | 0.24 | 0.28 | 0.25 | 0.28 |
| How frequently did you do the following prior to becoming pregnant?-Exercise: 5 | 0.07 | 0.12 | 0.07 | 0.12 | 0.07 | 0.12 | 0.06 | 0.12 | 0.07 | 0.11 |
| How frequently did you do the following prior to becoming pregnant?-Smoke marijuana: 1 | 0.03 | 0.05 | 0.03 | 0.05 | 0.03 | 0.06 | 0.03 | 0.05 | 0.03 | 0.05 |
| How frequently did you do the following prior to becoming pregnant?-Smoke marijuana: 2 | 0.02 | 0.03 | 0.03 | 0.02 | 0.03 | 0.02 | 0.03 | 0.03 | 0.03 | 0.03 |
| How frequently did you do the following prior to becoming pregnant?-Smoke marijuana: 3 | 0 | 0.02 | 0.01 | 0.02 | 0.01 | 0.02 | 0.01 | 0.02 | 0.01 | 0.01 |
| How frequently did you do the following prior to becoming pregnant?-Smoke marijuana: 4 | 0.05 | 0.05 | 0.05 | 0.06 | 0.05 | 0.06 | 0.06 | 0.05 | 0.06 | 0.04 |
| How frequently did you do the following prior to becoming pregnant?-Smoke marijuana: 5 | 0.89 | 0.84 | 0.88 | 0.85 | 0.88 | 0.85 | 0.88 | 0.86 | 0.88 | 0.87 |
| How frequently did you do the following prior to becoming pregnant?-Eat healthy: 1 | 0.36 | 0.24 | 0.35 | 0.27 | 0.34 | 0.27 | 0.35 | 0.28 | 0.34 | 0.28 |
| How frequently did you do the following prior to becoming pregnant?-Eat healthy: 2 | 0.49 | 0.51 | 0.49 | 0.51 | 0.5 | 0.49 | 0.51 | 0.49 | 0.51 | 0.47 |
| How frequently did you do the following prior to becoming pregnant?-Eat healthy: 3 | 0.08 | 0.12 | 0.09 | 0.1 | 0.09 | 0.1 | 0.09 | 0.1 | 0.09 | 0.1 |
| How frequently did you do the following prior to becoming pregnant?-Eat healthy: 4 | 0.06 | 0.11 | 0.07 | 0.09 | 0.06 | 0.12 | 0.06 | 0.11 | 0.06 | 0.11 |
| How frequently did you do the following prior to becoming pregnant?-Eat healthy: 5 | 0.01 | 0.02 | 0 | 0.03 | 0.01 | 0.03 | 0 | 0.03 | 0 | 0.03 |
| How frequently did you do the following prior to becoming pregnant?-Write in a journal or blog: 1 | 0.02 | 0.03 | 0.02 | 0.02 | 0.02 | 0.03 | 0.02 | 0.03 | 0.02 | 0.03 |
| How frequently did you do the following prior to becoming pregnant?-Write in a journal or blog: 2 | 0.06 | 0.04 | 0.07 | 0.04 | 0.06 | 0.04 | 0.06 | 0.05 | 0.06 | 0.05 |
| How frequently did you do the following prior to becoming pregnant?-Write in a journal or blog: 3 | 0.06 | 0.07 | 0.05 | 0.08 | 0.06 | 0.08 | 0.05 | 0.08 | 0.06 | 0.07 |
| How frequently did you do the following prior to becoming pregnant?-Write in a journal or blog: 4 | 0.21 | 0.2 | 0.22 | 0.19 | 0.21 | 0.2 | 0.22 | 0.19 | 0.22 | 0.2 |
| How frequently did you do the following prior to becoming pregnant?-Write in a journal or blog: 5 | 0.64 | 0.65 | 0.63 | 0.67 | 0.65 | 0.64 | 0.65 | 0.65 | 0.64 | 0.65 |
| How frequently did you do the following prior to becoming pregnant?-Use illegal drugs: 1 | 0 | 0.01 | 0 | 0.01 | 0 | 0.01 | 0 | 0.01 | 0 | 0.01 |
| How frequently did you do the following prior to becoming pregnant?-Use illegal drugs: 2 | 0 | 0.01 | 0 | 0.01 | 0 | 0.01 | 0 | 0.01 | 0 | 0.01 |
| How frequently did you do the following prior to becoming pregnant?-Use illegal drugs: 4 | 0.03 | 0.02 | 0.03 | 0.03 | 0.03 | 0.03 | 0.02 | 0.04 | 0.03 | 0.03 |
| How frequently did you do the following prior to becoming pregnant?-Use illegal drugs: 5 | 0.96 | 0.96 | 0.96 | 0.96 | 0.96 | 0.95 | 0.97 | 0.95 | 0.97 | 0.95 |
| How frequently did you do the following prior to becoming pregnant?-Practice yoga: 1 | 0.01 | 0.01 | 0.01 | 0.01 | 0.01 | 0.01 | 0.01 | 0.01 | 0.01 | 0.01 |
| How frequently did you do the following prior to becoming pregnant?-Practice yoga: 2 | 0.06 | 0.03 | 0.06 | 0.04 | 0.06 | 0.05 | 0.06 | 0.04 | 0.06 | 0.04 |
| How frequently did you do the following prior to becoming pregnant?-Practice yoga: 3 | 0.08 | 0.06 | 0.09 | 0.05 | 0.08 | 0.06 | 0.09 | 0.05 | 0.08 | 0.07 |
| How frequently did you do the following prior to becoming pregnant?-Practice yoga: 4 | 0.22 | 0.15 | 0.22 | 0.16 | 0.23 | 0.15 | 0.22 | 0.17 | 0.24 | 0.14 |
| How frequently did you do the following prior to becoming pregnant?-Practice yoga: 5 | 0.62 | 0.75 | 0.62 | 0.74 | 0.63 | 0.74 | 0.62 | 0.72 | 0.62 | 0.75 |
| Has a medical professional ever diagnosed you with any of the following conditions? (Please selec...-Allergies | 0.32 | 0.28 | 0.32 | 0.29 | 0.32 | 0.28 | 0.33 | 0.28 | 0.33 | 0.28 |
| Has a medical professional ever diagnosed you with any of the following conditions? (Please selec...-Substance addiction | 0 | 0.01 | 0 | 0.01 | 0 | 0.01 | NA | NA | NA | NA |
| Has a medical professional ever diagnosed you with any of the following conditions? (Please selec...-Substance addiction: -1 | NA | NA | NA | NA | NA | NA | 1 | 0.99 | 1 | 0.99 |
| Has a medical professional ever diagnosed you with any of the following conditions? (Please selec...-Other, please specify: | 0.1 | 0.1 | 0.09 | 0.12 | 0.09 | 0.11 | 0.09 | 0.11 | 0.09 | 0.11 |
| Has a medical professional ever diagnosed you with any of the following conditions? (Please selec...-None of these | 0.37 | 0.39 | 0.37 | 0.39 | 0.36 | 0.41 | 0.36 | 0.4 | 0.36 | 0.41 |
| Has a medical professional ever diagnosed you with any of the following conditions? (Please selec...-Endometriosis | 0.03 | 0.02 | 0.02 | 0.03 | 0.03 | 0.02 | 0.03 | 0.02 | 0.03 | 0.02 |
| Has a medical professional ever diagnosed you with any of the following conditions? (Please selec...-Diabetes (Type I or II) | 0.02 | 0.04 | 0.02 | 0.03 | 0.02 | 0.04 | 0.02 | 0.03 | 0.02 | 0.03 |
| Has a medical professional ever diagnosed you with any of the following conditions? (Please selec...-Fertility problems | 0.1 | 0.04 | 0.09 | 0.07 | 0.1 | 0.05 | 0.1 | 0.05 | 0.1 | 0.05 |
| Has a medical professional ever diagnosed you with any of the following conditions? (Please selec...-Fibromyalgia | 0.02 | 0.02 | 0.02 | 0.02 | 0.02 | 0.02 | 0.02 | 0.02 | 0.02 | 0.02 |
| Has a medical professional ever diagnosed you with any of the following conditions? (Please selec...-Hypertension (high blood pressure) | 0.04 | 0.06 | 0.04 | 0.06 | 0.05 | 0.05 | 0.05 | 0.05 | 0.05 | 0.05 |
| Has a medical professional ever diagnosed you with any of the following conditions? (Please selec...-Irritable bowel syndrome (IBS) | 0.04 | 0.04 | 0.04 | 0.04 | 0.05 | 0.03 | 0.04 | 0.04 | 0.05 | 0.03 |
| Has a medical professional ever diagnosed you with any of the following conditions? (Please selec...-Migraines | 0.16 | 0.16 | 0.17 | 0.15 | 0.17 | 0.14 | 0.16 | 0.16 | 0.17 | 0.16 |
| Has a medical professional ever diagnosed you with any of the following conditions? (Please selec...-Obesity | 0.12 | 0.11 | 0.12 | 0.12 | 0.13 | 0.11 | 0.13 | 0.1 | 0.13 | 0.1 |
| Has a medical professional ever diagnosed you with any of the following conditions? (Please selec...-Sleep disorders | 0.05 | 0.06 | 0.05 | 0.06 | 0.05 | 0.05 | 0.05 | 0.05 | 0.05 | 0.06 |
| Can you tell us, at any time prior to your pregnancy, had you suffered from any of the emotional...-Bipolar Disorder | 0.04 | 0.05 | 0.04 | 0.04 | 0.03 | 0.06 | 0.03 | 0.05 | 0.03 | 0.05 |
| Can you tell us, at any time prior to your pregnancy, had you suffered from any of the emotional...-None of these | 0.54 | 0.56 | 0.54 | 0.56 | 0.54 | 0.55 | 0.54 | 0.56 | 0.54 | 0.55 |
| Can you tell us, at any time prior to your pregnancy, had you suffered from any of the emotional...-Depression | 0.26 | 0.26 | 0.26 | 0.25 | 0.25 | 0.28 | 0.25 | 0.27 | 0.24 | 0.28 |
| Can you tell us, at any time prior to your pregnancy, had you suffered from any of the emotional...-Eating Disorder | 0.05 | 0.04 | 0.06 | 0.03 | 0.06 | 0.04 | 0.06 | 0.04 | 0.05 | 0.05 |
| Can you tell us, at any time prior to your pregnancy, had you suffered from any of the emotional...-Generalized Anxiety Disorder | 0.16 | 0.15 | 0.17 | 0.14 | 0.16 | 0.16 | 0.17 | 0.15 | 0.16 | 0.15 |
| Can you tell us, at any time prior to your pregnancy, had you suffered from any of the emotional...-Obsessive Compulsive Disorder (OCD) | 0.04 | 0.02 | 0.04 | 0.02 | 0.04 | 0.02 | 0.03 | 0.04 | 0.04 | 0.03 |
| Can you tell us, at any time prior to your pregnancy, had you suffered from any of the emotional...-Panic attacks | 0.14 | 0.17 | 0.16 | 0.14 | 0.14 | 0.17 | 0.15 | 0.15 | 0.14 | 0.16 |
| Can you tell us, at any time prior to your pregnancy, had you suffered from any of the emotional...-Postpartum Depression | 0.09 | 0.06 | 0.08 | 0.07 | 0.09 | 0.05 | 0.1 | 0.05 | 0.09 | 0.06 |
| Can you tell us, at any time prior to your pregnancy, had you suffered from any of the emotional...-Post-Traumatic Stress Disorder (PTSD) | 0.06 | 0.09 | 0.06 | 0.09 | 0.07 | 0.08 | 0.07 | 0.08 | 0.07 | 0.08 |
| Can you tell us, at any time prior to your pregnancy, had you suffered from any of the emotional...-Other emotional/mental health disorders | 0.03 | 0.04 | 0.02 | 0.05 | 0.03 | 0.05 | 0.03 | 0.04 | 0.03 | 0.04 |
| Prior to this pregnancy, were you taking any of the following types of prescription medications f...-Antidepressants | 0.12 | 0.1 | 0.11 | 0.11 | 0.11 | 0.12 | 0.11 | 0.11 | 0.1 | 0.12 |
| Prior to this pregnancy, were you taking any of the following types of prescription medications f...-Anti-anxiety medications | 0.09 | 0.09 | 0.09 | 0.09 | 0.08 | 0.1 | 0.09 | 0.08 | 0.1 | 0.08 |
| Prior to this pregnancy, were you taking any of the following types of prescription medications f...-Sleep medications | 0.06 | 0.06 | 0.07 | 0.06 | 0.06 | 0.07 | 0.06 | 0.07 | 0.06 | 0.08 |
| Prior to this pregnancy, were you taking any of the following types of prescription medications f...-Other, please specify: | 0.03 | 0.03 | 0.02 | 0.03 | 0.03 | 0.03 | 0.03 | 0.03 | 0.03 | 0.03 |
| Prior to this pregnancy, were you taking any of the following types of prescription medications f...-None of these | 0.76 | 0.79 | 0.77 | 0.78 | 0.77 | 0.78 | 0.77 | 0.78 | 0.77 | 0.77 |
| Prior to this pregnancy, were you taking any of the following types of prescription medications f...-Prefer not to say | 0.01 | 0.02 | 0.01 | 0.02 | 0.01 | 0.02 | 0.01 | 0.02 | 0.01 | 0.03 |
| Which of the following, if any, has happened to you within the past year?¬† (Please select all tha...-Death in family or of a close friend | 0.28 | 0.31 | 0.29 | 0.3 | 0.29 | 0.28 | 0.29 | 0.29 | 0.3 | 0.27 |
| Which of the following, if any, has happened to you within the past year?¬† (Please select all tha...-Victim of a non-violent crime (identity theft, burglary, etc.) | 0.04 | 0.03 | 0.04 | 0.04 | 0.05 | 0.03 | 0.05 | 0.03 | 0.04 | 0.03 |
| Which of the following, if any, has happened to you within the past year?¬† (Please select all tha...-Victim of a violent crime | 0.01 | 0.03 | 0.01 | 0.02 | 0.01 | 0.03 | 0.01 | 0.03 | 0.01 | 0.03 |
| Which of the following, if any, has happened to you within the past year?¬† (Please select all tha...-None of these | 0.29 | 0.23 | 0.28 | 0.25 | 0.28 | 0.25 | 0.28 | 0.26 | 0.28 | 0.26 |
| Which of the following, if any, has happened to you within the past year?¬† (Please select all tha...-Divorced, separated or ended a relationship | 0.08 | 0.13 | 0.09 | 0.12 | 0.08 | 0.14 | 0.07 | 0.13 | 0.07 | 0.13 |
| Which of the following, if any, has happened to you within the past year?¬† (Please select all tha...-Financial hardship | 0.31 | 0.37 | 0.31 | 0.35 | 0.31 | 0.37 | 0.3 | 0.37 | 0.31 | 0.35 |
| Which of the following, if any, has happened to you within the past year?¬† (Please select all tha...-Legal problem/litigation | 0.05 | 0.06 | 0.05 | 0.05 | 0.05 | 0.05 | 0.05 | 0.05 | 0.05 | 0.05 |
| Which of the following, if any, has happened to you within the past year?¬† (Please select all tha...-Had a miscarriage | 0.14 | 0.13 | 0.14 | 0.12 | 0.13 | 0.13 | 0.14 | 0.12 | 0.14 | 0.12 |
| Which of the following, if any, has happened to you within the past year?¬† (Please select all tha...-Moved to a new location | 0.28 | 0.25 | 0.28 | 0.24 | 0.27 | 0.25 | 0.27 | 0.26 | 0.27 | 0.26 |
| Which of the following, if any, has happened to you within the past year?¬† (Please select all tha...-Personally lost a job | 0.11 | 0.14 | 0.1 | 0.15 | 0.11 | 0.14 | 0.1 | 0.14 | 0.1 | 0.14 |
| Which of the following, if any, has happened to you within the past year?¬† (Please select all tha...-Spouse/partner lost a job | 0.09 | 0.08 | 0.09 | 0.08 | 0.08 | 0.08 | 0.1 | 0.07 | 0.09 | 0.07 |
| Which of the following, if any, has happened to you within the past year?¬† (Please select all tha...-Victim of a natural disaster | 0.01 | 0.01 | 0.01 | 0.01 | 0.01 | 0.01 | 0.01 | 0.01 | 0.01 | 0.01 |
| Do you have any children?: No | 0.41 | 0.41 | 0.41 | 0.4 | 0.4 | 0.42 | 0.39 | 0.43 | 0.39 | 0.43 |
| Do you have any children?: Yes | 0.59 | 0.59 | 0.59 | 0.6 | 0.6 | 0.58 | 0.61 | 0.57 | 0.61 | 0.57 |
| What are the ages of your children? (Please select all that apply.)-Less than 1 year old | 0.05 | 0.04 | 0.04 | 0.05 | 0.05 | 0.04 | 0.04 | 0.05 | 0.04 | 0.05 |
| What are the ages of your children? (Please select all that apply.)-1-2 years old | 0.25 | 0.18 | 0.25 | 0.18 | 0.25 | 0.18 | 0.27 | 0.16 | 0.26 | 0.17 |
| What are the ages of your children? (Please select all that apply.)-3-5 years old | 0.24 | 0.22 | 0.23 | 0.24 | 0.24 | 0.23 | 0.24 | 0.23 | 0.23 | 0.24 |
| What are the ages of your children? (Please select all that apply.)-6-12 years old | 0.23 | 0.27 | 0.22 | 0.29 | 0.24 | 0.27 | 0.23 | 0.27 | 0.24 | 0.26 |
| What are the ages of your children? (Please select all that apply.)-13-17 years old | 0.07 | 0.11 | 0.06 | 0.11 | 0.07 | 0.1 | 0.08 | 0.08 | 0.08 | 0.08 |
| What are the ages of your children? (Please select all that apply.)-18 years old or older | 0.03 | 0.04 | 0.03 | 0.04 | 0.03 | 0.04 | 0.03 | 0.04 | 0.03 | 0.04 |
| What is your current marital status?¬†¬†: Divorced | 0.01 | 0.02 | 0.02 | 0.02 | 0.01 | 0.02 | 0.01 | 0.02 | 0.01 | 0.02 |
| What is your current marital status?¬†¬†: Living with a partner | 0.17 | 0.28 | 0.18 | 0.26 | 0.18 | 0.27 | 0.16 | 0.26 | 0.17 | 0.27 |
| What is your current marital status?¬†¬†: Married | 0.68 | 0.44 | 0.67 | 0.48 | 0.68 | 0.43 | 0.7 | 0.47 | 0.68 | 0.46 |
| What is your current marital status?¬†¬†: Other | 0.01 | 0.03 | 0.01 | 0.03 | 0.01 | 0.03 | 0.01 | 0.03 | 0.01 | 0.03 |
| What is your current marital status?¬†¬†: Single | 0.12 | 0.23 | 0.13 | 0.22 | 0.12 | 0.24 | 0.11 | 0.22 | 0.12 | 0.22 |
| Which of the following best describes your current employment status?: Employed and currently working full-time | 0.54 | 0.49 | 0.53 | 0.51 | 0.52 | 0.52 | 0.52 | 0.53 | 0.53 | 0.51 |
| Which of the following best describes your current employment status?: Employed and currently working part-time | 0.11 | 0.15 | 0.1 | 0.15 | 0.11 | 0.14 | 0.1 | 0.14 | 0.11 | 0.14 |
| Which of the following best describes your current employment status?: Other | 0.02 | 0.02 | 0.02 | 0.02 | 0.02 | 0.02 | 0.02 | 0.02 | 0.02 | 0.02 |
| Which of the following best describes your current employment status?: Stay at home mom | 0.21 | 0.18 | 0.23 | 0.15 | 0.23 | 0.15 | 0.24 | 0.14 | 0.22 | 0.16 |
| Which of the following best describes your current employment status?: Student | 0.06 | 0.05 | 0.06 | 0.05 | 0.06 | 0.05 | 0.05 | 0.06 | 0.05 | 0.06 |
| Which of the following best describes your current employment status?: Unemployed | 0.06 | 0.12 | 0.06 | 0.12 | 0.06 | 0.13 | 0.07 | 0.1 | 0.06 | 0.12 |
| Does your spouse or partner suffer from any of the emotional or mental health conditions noted be...-Bipolar Disorder | 0.03 | 0.02 | 0.03 | 0.02 | 0.02 | 0.03 | 0.02 | 0.03 | 0.02 | 0.03 |
| Does your spouse or partner suffer from any of the emotional or mental health conditions noted be...-Depression | 0.07 | 0.06 | 0.06 | 0.08 | 0.06 | 0.07 | 0.06 | 0.08 | 0.06 | 0.07 |
| Does your spouse or partner suffer from any of the emotional or mental health conditions noted be...-Eating Disorder | NA | NA | 0 | 0 | 0 | 0 | 0 | 0 | 0 | 0 |
| Does your spouse or partner suffer from any of the emotional or mental health conditions noted be...-Eating Disorder: -1 | 1 | 0.99 | NA | NA | NA | NA | NA | NA | NA | NA |
| Does your spouse or partner suffer from any of the emotional or mental health conditions noted be...-Generalized Anxiety Disorder | 0.04 | 0.03 | 0.05 | 0.02 | 0.04 | 0.03 | 0.05 | 0.03 | 0.05 | 0.02 |
| Does your spouse or partner suffer from any of the emotional or mental health conditions noted be...-Obsessive Compulsive Disorder (OCD) | 0.01 | 0.02 | 0.01 | 0.02 | 0.01 | 0.02 | 0.01 | 0.01 | 0.01 | 0.01 |
| Does your spouse or partner suffer from any of the emotional or mental health conditions noted be...-Panic attacks | 0.02 | 0.01 | 0.02 | 0.01 | 0.01 | 0.02 | 0.01 | 0.02 | 0.01 | 0.01 |
| Does your spouse or partner suffer from any of the emotional or mental health conditions noted be...-Post-Traumatic Stress Disorder (PTSD) | 0.03 | 0.03 | 0.02 | 0.03 | 0.03 | 0.03 | 0.03 | 0.03 | 0.03 | 0.02 |
| Does your spouse or partner suffer from any of the emotional or mental health conditions noted be...-Other emotional/mental health disorders | 0.03 | 0.03 | 0.02 | 0.03 | 0.03 | 0.03 | 0.02 | 0.03 | 0.03 | 0.03 |
| Does your spouse or partner suffer from any of the emotional or mental health conditions noted be...-None of these | 0.66 | 0.54 | 0.66 | 0.56 | 0.67 | 0.53 | 0.68 | 0.55 | 0.66 | 0.56 |
| What is the highest level of education you have completed?: 1 | 0.03 | 0.06 | 0.03 | 0.06 | 0.03 | 0.06 | 0.03 | 0.05 | 0.03 | 0.06 |
| What is the highest level of education you have completed?: 2 | 0.11 | 0.21 | 0.11 | 0.21 | 0.11 | 0.22 | 0.12 | 0.19 | 0.11 | 0.2 |
| What is the highest level of education you have completed?: 3 | 0.31 | 0.37 | 0.31 | 0.35 | 0.31 | 0.37 | 0.3 | 0.36 | 0.3 | 0.36 |
| What is the highest level of education you have completed?: 4 | 0.33 | 0.23 | 0.32 | 0.25 | 0.33 | 0.23 | 0.33 | 0.25 | 0.33 | 0.23 |
| What is the highest level of education you have completed?: 5 | 0.05 | 0.02 | 0.05 | 0.01 | 0.05 | 0.02 | 0.05 | 0.02 | 0.05 | 0.02 |
| What is the highest level of education you have completed?: 6 | 0.17 | 0.1 | 0.17 | 0.1 | 0.17 | 0.1 | 0.17 | 0.11 | 0.17 | 0.1 |
| What is the highest level of education you have completed?: 7 | 0.01 | 0.01 | 0 | 0.01 | 0 | 0.01 | 0.01 | 0.01 | 0.01 | 0.01 |
| What is your ethnic background? (Please select all that apply)-African American | 0.14 | 0.24 | 0.14 | 0.22 | 0.14 | 0.24 | 0.13 | 0.22 | 0.14 | 0.22 |
| What is your ethnic background? (Please select all that apply)-Asian /Pacific Islander | 0.05 | 0.04 | 0.05 | 0.04 | 0.06 | 0.03 | 0.05 | 0.05 | 0.05 | 0.05 |
| What is your ethnic background? (Please select all that apply)-Caucasian | 0.62 | 0.47 | 0.62 | 0.48 | 0.62 | 0.48 | 0.64 | 0.48 | 0.63 | 0.48 |
| What is your ethnic background? (Please select all that apply)-Hispanic | 0.18 | 0.2 | 0.17 | 0.21 | 0.17 | 0.2 | 0.16 | 0.21 | 0.16 | 0.22 |
| What is your ethnic background? (Please select all that apply)-Native American | 0.03 | 0.06 | 0.04 | 0.05 | 0.04 | 0.06 | 0.03 | 0.06 | 0.03 | 0.07 |
| What is your ethnic background? (Please select all that apply)-Other | 0.03 | 0.04 | 0.04 | 0.03 | 0.04 | 0.03 | 0.04 | 0.03 | 0.04 | 0.03 |
| What is your ethnic background? (Please select all that apply)-Prefer not to answer | 0.02 | 0.05 | 0.02 | 0.05 | 0.03 | 0.04 | 0.02 | 0.04 | 0.03 | 0.04 |
| What is your annual household income before taxes?: 1 | 0.2 | 0.29 | 0.2 | 0.28 | 0.19 | 0.31 | 0.19 | 0.29 | 0.19 | 0.3 |
| What is your annual household income before taxes?: 2 | 0.13 | 0.15 | 0.13 | 0.15 | 0.13 | 0.16 | 0.12 | 0.16 | 0.13 | 0.15 |
| What is your annual household income before taxes?: 3 | 0.09 | 0.14 | 0.11 | 0.1 | 0.11 | 0.11 | 0.11 | 0.11 | 0.11 | 0.1 |
| What is your annual household income before taxes?: 4 | 0.16 | 0.12 | 0.15 | 0.14 | 0.16 | 0.12 | 0.16 | 0.12 | 0.17 | 0.11 |
| What is your annual household income before taxes?: 5 | 0.13 | 0.09 | 0.12 | 0.11 | 0.13 | 0.09 | 0.12 | 0.11 | 0.13 | 0.1 |
| What is your annual household income before taxes?: 6 | 0.1 | 0.03 | 0.1 | 0.05 | 0.1 | 0.04 | 0.11 | 0.05 | 0.1 | 0.05 |
| What is your annual household income before taxes?: 7 | 0.12 | 0.06 | 0.12 | 0.07 | 0.12 | 0.07 | 0.12 | 0.08 | 0.11 | 0.08 |
| What is your annual household income before taxes?: 8 | 0.06 | 0.12 | 0.07 | 0.11 | 0.07 | 0.12 | 0.07 | 0.1 | 0.07 | 0.11 |

## Discriminative performance of the models predicting dropout

| outcome | Variable type | Number of features | AUROC |
| --- | --- | --- | --- |
| Trimester 1 | 1 | 17 | 0.54960221 |
| Trimester 1 | 2 | 21 | 0.54880576 |
| Trimester 1 | 3 | 164 | 0.65328081 |
| Trimester 2 | 1 | 17 | 0.54228141 |
| Trimester 2 | 2 | 21 | 0.5496205 |
| Trimester 2 | 3 | 164 | 0.63042266 |
| Trimester 3 | 1 | 17 | 0.57389249 |
| Trimester 3 | 2 | 21 | 0.57340449 |
| Trimester 3 | 3 | 164 | 0.65308619 |
| After 1 | 1 | 17 | 0.57133528 |
| After 1 | 2 | 21 | 0.56509373 |
| After 1 | 3 | 164 | 0.66456677 |
| After 2 | 1 | 17 | 0.57192318 |
| After 2 | 2 | 21 | 0.57175625 |
| After 2 | 3 | 164 | 0.64912474 |
