# Supplementary material for: Development of multivariable models to predict perinatal depression before and after delivery using patient reported survey responses at weeks 4–10 of pregnancy
Source: BMC Pregnancy Childbirth. 2022 May 26;22:442. doi: 10.1186/s12884-022-04741-9 (PMC9137134; doi:10.1186/s12884-022-04741-9)
Supplement: Supplementary file 2 — Additional file 2. Characteristics of people with and without the outcomes. A word document containing a table with the mean values for each of the baseline survey for EPDS/GAD/PRES for those who had an EPDS < 12 vs an EPDS >= 12 for each outcome survey. [file 12884_2022_4741_MOESM2_ESM.docx]

Additional file 2: Characteristics of people with and without the outcomes

|  |  | Trimester 1 | | Trimester 2 | | Trimester 3 | | After Delievery 1 | | After Delievery 2 | |
| --- | --- | --- | --- | --- | --- | --- | --- | --- | --- | --- | --- |
| Covariate Name | Survey Questions: Response | EPDS < 12 | EPDS >= 12 | EPDS < 12 | EPDS >= 12 | EPDS < 12 | EPDS >= 12 | EPDS < 12 | EPDS >= 12 | EPDS < 12 | EPDS >= 12 |
| EPDS_B_1 | I have been able to laugh and see the funny side of things.: 1 | 0.85 | 0.55 | 0.82 | 0.63 | 0.82 | 0.69 | 0.82 | 0.66 | 0.81 | 0.67 |
| EPDS_B_1 | I have been able to laugh and see the funny side of things.: 2 | 0.13 | 0.34 | 0.15 | 0.3 | 0.16 | 0.23 | 0.16 | 0.29 | 0.15 | 0.3 |
| EPDS_B_1 | I have been able to laugh and see the funny side of things.: 3 | 0.02 | 0.08 | 0.02 | 0.05 | 0.01 | 0.07 | 0.02 | 0.04 | 0.02 | 0.02 |
| EPDS_B_1 | I have been able to laugh and see the funny side of things.: 4 | 0 | 0.03 | 0.01 | 0.02 | 0.01 | 0.01 | 0.01 | 0.01 | NA | NA |
| EPDS_B_2 | I have looked forward with enjoyment to things.: 1 | 0.82 | 0.49 | 0.78 | 0.64 | 0.8 | 0.64 | 0.81 | 0.6 | 0.8 | 0.6 |
| EPDS_B_2 | I have looked forward with enjoyment to things.: 2 | 0.15 | 0.41 | 0.19 | 0.24 | 0.17 | 0.3 | 0.16 | 0.34 | 0.16 | 0.36 |
| EPDS_B_2 | I have looked forward with enjoyment to things.: 3 | 0.03 | 0.08 | 0.02 | 0.1 | 0.03 | 0.05 | 0.03 | 0.06 | 0.03 | 0.04 |
| EPDS_B_2 | I have looked forward with enjoyment to things.: 4 | 0 | 0.03 | 0.01 | 0.02 | 0.01 | 0.01 | NA | NA | NA | NA |
| EPDS_B_3 | I have blamed myself unnecessarily when things went wrong.: 1 | 0.27 | 0.13 | 0.26 | 0.14 | 0.28 | 0.11 | 0.27 | 0.1 | 0.26 | 0.13 |
| EPDS_B_3 | I have blamed myself unnecessarily when things went wrong.: 2 | 0.43 | 0.33 | 0.43 | 0.34 | 0.44 | 0.37 | 0.42 | 0.47 | 0.42 | 0.42 |
| EPDS_B_3 | I have blamed myself unnecessarily when things went wrong.: 3 | 0.27 | 0.43 | 0.27 | 0.41 | 0.24 | 0.42 | 0.27 | 0.35 | 0.28 | 0.36 |
| EPDS_B_3 | I have blamed myself unnecessarily when things went wrong.: 4 | 0.03 | 0.11 | 0.04 | 0.12 | 0.04 | 0.1 | 0.04 | 0.08 | 0.04 | 0.09 |
| EPDS_B_4 | I have been anxious or worried for no good reason.: 1 | 0.24 | 0.06 | 0.24 | 0.09 | 0.24 | 0.09 | 0.24 | 0.05 | 0.23 | 0.09 |
| EPDS_B_4 | I have been anxious or worried for no good reason.: 2 | 0.29 | 0.16 | 0.28 | 0.2 | 0.3 | 0.18 | 0.3 | 0.17 | 0.27 | 0.21 |
| EPDS_B_4 | I have been anxious or worried for no good reason.: 3 | 0.45 | 0.56 | 0.44 | 0.55 | 0.41 | 0.59 | 0.43 | 0.61 | 0.44 | 0.61 |
| EPDS_B_4 | I have been anxious or worried for no good reason.: 4 | 0.03 | 0.22 | 0.05 | 0.16 | 0.05 | 0.14 | 0.04 | 0.17 | 0.05 | 0.09 |
| EPDS_B_5 | I have felt scared or panicky for no very good reason.: 1 | 0.39 | 0.14 | 0.38 | 0.13 | 0.4 | 0.15 | 0.38 | 0.14 | 0.37 | 0.16 |
| EPDS_B_5 | I have felt scared or panicky for no very good reason.: 2 | 0.33 | 0.24 | 0.3 | 0.32 | 0.32 | 0.31 | 0.32 | 0.27 | 0.31 | 0.33 |
| EPDS_B_5 | I have felt scared or panicky for no very good reason.: 3 | 0.26 | 0.51 | 0.29 | 0.47 | 0.27 | 0.45 | 0.26 | 0.52 | 0.28 | 0.48 |
| EPDS_B_5 | I have felt scared or panicky for no very good reason.: 4 | 0.01 | 0.11 | 0.03 | 0.08 | 0.02 | 0.09 | 0.04 | 0.06 | 0.04 | 0.03 |
| EPDS_B_6 | Things have been getting on top of me.: 1 | 0.28 | 0.09 | 0.28 | 0.1 | 0.3 | 0.09 | 0.28 | 0.08 | 0.28 | 0.07 |
| EPDS_B_6 | Things have been getting on top of me.: 2 | 0.51 | 0.39 | 0.48 | 0.45 | 0.47 | 0.46 | 0.47 | 0.48 | 0.46 | 0.54 |
| EPDS_B_6 | Things have been getting on top of me.: 3 | 0.19 | 0.43 | 0.23 | 0.37 | 0.21 | 0.39 | 0.22 | 0.4 | 0.24 | 0.34 |
| EPDS_B_6 | Things have been getting on top of me.: 4 | 0.02 | 0.09 | 0.01 | 0.08 | 0.01 | 0.06 | 0.03 | 0.04 | 0.02 | 0.06 |
| EPDS_B_7 | I have been so unhappy that I have had difficulty sleeping.: 1 | 0.74 | 0.44 | 0.74 | 0.45 | 0.73 | 0.49 | 0.73 | 0.51 | 0.73 | 0.44 |
| EPDS_B_7 | I have been so unhappy that I have had difficulty sleeping.: 2 | 0.19 | 0.23 | 0.19 | 0.27 | 0.18 | 0.24 | 0.19 | 0.23 | 0.2 | 0.26 |
| EPDS_B_7 | I have been so unhappy that I have had difficulty sleeping.: 3 | 0.05 | 0.24 | 0.05 | 0.22 | 0.06 | 0.22 | 0.06 | 0.22 | 0.05 | 0.26 |
| EPDS_B_7 | I have been so unhappy that I have had difficulty sleeping.: 4 | 0.02 | 0.09 | 0.02 | 0.06 | 0.03 | 0.04 | 0.03 | 0.04 | 0.03 | 0.04 |
| EPDS_B_8 | I have felt sad or miserable.: 1 | 0.61 | 0.26 | 0.6 | 0.3 | 0.61 | 0.33 | 0.59 | 0.32 | 0.59 | 0.31 |
| EPDS_B_8 | I have felt sad or miserable.: 2 | 0.27 | 0.36 | 0.28 | 0.35 | 0.27 | 0.36 | 0.29 | 0.39 | 0.29 | 0.36 |
| EPDS_B_8 | I have felt sad or miserable.: 3 | 0.12 | 0.3 | 0.12 | 0.28 | 0.11 | 0.28 | 0.11 | 0.25 | 0.11 | 0.3 |
| EPDS_B_8 | I have felt sad or miserable.: 4 | 0.01 | 0.08 | 0.01 | 0.07 | 0.01 | 0.04 | 0.01 | 0.04 | 0.01 | 0.02 |
| EPDS_B_9 | I have been so unhappy that I have been crying.: 1 | 0.71 | 0.33 | 0.71 | 0.34 | 0.72 | 0.41 | 0.69 | 0.44 | 0.68 | 0.43 |
| EPDS_B_9 | I have been so unhappy that I have been crying.: 2 | 0.27 | 0.47 | 0.27 | 0.48 | 0.26 | 0.46 | 0.27 | 0.43 | 0.27 | 0.47 |
| EPDS_B_9 | I have been so unhappy that I have been crying.: 3 | 0.01 | 0.16 | 0.03 | 0.14 | 0.02 | 0.11 | 0.02 | 0.13 | 0.03 | 0.08 |
| EPDS_B_9 | I have been so unhappy that I have been crying.: 4 | 0 | 0.03 | 0 | 0.05 | 0.01 | 0.02 | NA | NA | 0.01 | 0.02 |
| GAD_B_1 | Thinking about how you‚Äôve been feeling more recently, over the last 2 weeks, how often have you b...-Feeling nervous, anxious, or on edge: 1 | 0.47 | 0.18 | 0.45 | 0.21 | 0.47 | 0.21 | 0.48 | 0.16 | 0.46 | 0.16 |
| GAD_B_1 | Thinking about how you‚Äôve been feeling more recently, over the last 2 weeks, how often have you b...-Feeling nervous, anxious, or on edge: 2 | 0.37 | 0.44 | 0.39 | 0.46 | 0.38 | 0.45 | 0.36 | 0.51 | 0.36 | 0.52 |
| GAD_B_1 | Thinking about how you‚Äôve been feeling more recently, over the last 2 weeks, how often have you b...-Feeling nervous, anxious, or on edge: 3 | 0.11 | 0.22 | 0.11 | 0.18 | 0.11 | 0.19 | 0.1 | 0.21 | 0.11 | 0.22 |
| GAD_B_1 | Thinking about how you‚Äôve been feeling more recently, over the last 2 weeks, how often have you b...-Feeling nervous, anxious, or on edge: 4 | 0.04 | 0.16 | 0.05 | 0.15 | 0.04 | 0.15 | 0.06 | 0.13 | 0.07 | 0.1 |
| GAD_B_2 | Thinking about how you‚Äôve been feeling more recently, over the last 2 weeks, how often have you b...-Not being able to stop or control worrying: 1 | 0.6 | 0.33 | 0.61 | 0.32 | 0.63 | 0.28 | 0.64 | 0.25 | 0.62 | 0.29 |
| GAD_B_2 | Thinking about how you‚Äôve been feeling more recently, over the last 2 weeks, how often have you b...-Not being able to stop or control worrying: 2 | 0.29 | 0.28 | 0.28 | 0.34 | 0.25 | 0.4 | 0.25 | 0.42 | 0.25 | 0.43 |
| GAD_B_2 | Thinking about how you‚Äôve been feeling more recently, over the last 2 weeks, how often have you b...-Not being able to stop or control worrying: 3 | 0.09 | 0.26 | 0.08 | 0.2 | 0.09 | 0.21 | 0.07 | 0.23 | 0.09 | 0.18 |
| GAD_B_2 | Thinking about how you‚Äôve been feeling more recently, over the last 2 weeks, how often have you b...-Not being able to stop or control worrying: 4 | 0.03 | 0.13 | 0.03 | 0.14 | 0.02 | 0.11 | 0.04 | 0.1 | 0.04 | 0.1 |
| GAD_B_3 | Thinking about how you‚Äôve been feeling more recently, over the last 2 weeks, how often have you b...-Worrying too much about different things: 1 | 0.49 | 0.18 | 0.49 | 0.21 | 0.51 | 0.19 | 0.51 | 0.14 | 0.48 | 0.2 |
| GAD_B_3 | Thinking about how you‚Äôve been feeling more recently, over the last 2 weeks, how often have you b...-Worrying too much about different things: 2 | 0.37 | 0.42 | 0.38 | 0.42 | 0.36 | 0.46 | 0.35 | 0.48 | 0.36 | 0.47 |
| GAD_B_3 | Thinking about how you‚Äôve been feeling more recently, over the last 2 weeks, how often have you b...-Worrying too much about different things: 3 | 0.1 | 0.26 | 0.09 | 0.23 | 0.09 | 0.23 | 0.09 | 0.29 | 0.11 | 0.22 |
| GAD_B_3 | Thinking about how you‚Äôve been feeling more recently, over the last 2 weeks, how often have you b...-Worrying too much about different things: 4 | 0.05 | 0.14 | 0.04 | 0.14 | 0.04 | 0.12 | 0.05 | 0.09 | 0.05 | 0.1 |
| GAD_B_4 | Thinking about how you‚Äôve been feeling more recently, over the last 2 weeks, how often have you b...-Trouble relaxing: 1 | 0.51 | 0.22 | 0.52 | 0.22 | 0.54 | 0.21 | 0.51 | 0.22 | 0.49 | 0.27 |
| GAD_B_4 | Thinking about how you‚Äôve been feeling more recently, over the last 2 weeks, how often have you b...-Trouble relaxing: 2 | 0.37 | 0.46 | 0.34 | 0.46 | 0.33 | 0.51 | 0.34 | 0.53 | 0.36 | 0.48 |
| GAD_B_4 | Thinking about how you‚Äôve been feeling more recently, over the last 2 weeks, how often have you b...-Trouble relaxing: 3 | 0.09 | 0.17 | 0.11 | 0.17 | 0.09 | 0.16 | 0.1 | 0.16 | 0.11 | 0.17 |
| GAD_B_4 | Thinking about how you‚Äôve been feeling more recently, over the last 2 weeks, how often have you b...-Trouble relaxing: 4 | 0.03 | 0.16 | 0.03 | 0.15 | 0.03 | 0.12 | 0.05 | 0.09 | 0.05 | 0.08 |
| GAD_B_5 | Thinking about how you‚Äôve been feeling more recently, over the last 2 weeks, how often have you b...-Being so restless that it's hard to sit still: 1 | 0.74 | 0.43 | 0.73 | 0.5 | 0.75 | 0.49 | 0.72 | 0.51 | 0.72 | 0.53 |
| GAD_B_5 | Thinking about how you‚Äôve been feeling more recently, over the last 2 weeks, how often have you b...-Being so restless that it's hard to sit still: 2 | 0.17 | 0.28 | 0.19 | 0.25 | 0.16 | 0.32 | 0.17 | 0.31 | 0.18 | 0.28 |
| GAD_B_5 | Thinking about how you‚Äôve been feeling more recently, over the last 2 weeks, how often have you b...-Being so restless that it's hard to sit still: 3 | 0.06 | 0.19 | 0.06 | 0.14 | 0.06 | 0.11 | 0.07 | 0.13 | 0.06 | 0.15 |
| GAD_B_5 | Thinking about how you‚Äôve been feeling more recently, over the last 2 weeks, how often have you b...-Being so restless that it's hard to sit still: 4 | 0.03 | 0.09 | 0.02 | 0.12 | 0.03 | 0.07 | 0.04 | 0.05 | 0.04 | 0.04 |
| GAD_B_6 | Thinking about how you‚Äôve been feeling more recently, over the last 2 weeks, how often have you b...-Becoming easily annoyed or irritable: 1 | 0.26 | 0.09 | 0.26 | 0.12 | 0.27 | 0.09 | 0.26 | 0.1 | 0.26 | 0.09 |
| GAD_B_6 | Thinking about how you‚Äôve been feeling more recently, over the last 2 weeks, how often have you b...-Becoming easily annoyed or irritable: 2 | 0.48 | 0.41 | 0.47 | 0.44 | 0.47 | 0.45 | 0.46 | 0.44 | 0.46 | 0.47 |
| GAD_B_6 | Thinking about how you‚Äôve been feeling more recently, over the last 2 weeks, how often have you b...-Becoming easily annoyed or irritable: 3 | 0.19 | 0.22 | 0.18 | 0.21 | 0.17 | 0.26 | 0.2 | 0.21 | 0.18 | 0.26 |
| GAD_B_6 | Thinking about how you‚Äôve been feeling more recently, over the last 2 weeks, how often have you b...-Becoming easily annoyed or irritable: 4 | 0.07 | 0.29 | 0.08 | 0.23 | 0.09 | 0.19 | 0.08 | 0.25 | 0.1 | 0.18 |
| GAD_B_7 | Thinking about how you‚Äôve been feeling more recently, over the last 2 weeks, how often have you b...-Feeling afraid as if something awful might happen: 1 | 0.57 | 0.27 | 0.55 | 0.3 | 0.55 | 0.29 | 0.55 | 0.27 | 0.55 | 0.24 |
| GAD_B_7 | Thinking about how you‚Äôve been feeling more recently, over the last 2 weeks, how often have you b...-Feeling afraid as if something awful might happen: 2 | 0.3 | 0.3 | 0.29 | 0.34 | 0.29 | 0.36 | 0.29 | 0.34 | 0.29 | 0.44 |
| GAD_B_7 | Thinking about how you‚Äôve been feeling more recently, over the last 2 weeks, how often have you b...-Feeling afraid as if something awful might happen: 3 | 0.08 | 0.23 | 0.1 | 0.14 | 0.11 | 0.16 | 0.11 | 0.17 | 0.11 | 0.17 |
| GAD_B_7 | Thinking about how you‚Äôve been feeling more recently, over the last 2 weeks, how often have you b...-Feeling afraid as if something awful might happen: 4 | 0.05 | 0.2 | 0.05 | 0.22 | 0.04 | 0.19 | 0.05 | 0.22 | 0.06 | 0.16 |
| GAD_B_8 | Thinking about how you‚Äôve been feeling more recently, over the last 2 weeks, how often have you b...-Feeling I might lose control of myself: 1 | 0.86 | 0.54 | 0.85 | 0.59 | 0.85 | 0.64 | 0.85 | 0.64 | 0.85 | 0.65 |
| GAD_B_8 | Thinking about how you‚Äôve been feeling more recently, over the last 2 weeks, how often have you b...-Feeling I might lose control of myself: 2 | 0.1 | 0.27 | 0.11 | 0.25 | 0.1 | 0.24 | 0.1 | 0.26 | 0.1 | 0.26 |
| GAD_B_8 | Thinking about how you‚Äôve been feeling more recently, over the last 2 weeks, how often have you b...-Feeling I might lose control of myself: 3 | 0.03 | 0.09 | 0.03 | 0.08 | 0.04 | 0.06 | 0.02 | 0.06 | 0.03 | 0.06 |
| GAD_B_8 | Thinking about how you‚Äôve been feeling more recently, over the last 2 weeks, how often have you b...-Feeling I might lose control of myself: 4 | 0.02 | 0.09 | 0.01 | 0.07 | 0.02 | 0.06 | 0.02 | 0.04 | 0.02 | 0.03 |
| PR_ES_B_1 | How often have you felt the sentiment stated in each of the sentences below?-I have someone who will listen to me when I need to talk: 1 | 0 | 0.02 | NA | NA | NA | NA | NA | NA | 0 | 0.01 |
| PR_ES_B_1 | How often have you felt the sentiment stated in each of the sentences below?-I have someone who will listen to me when I need to talk: 2 | 0.02 | 0.09 | 0.02 | 0.08 | 0.02 | 0.06 | 0.03 | 0.05 | 0.03 | 0.06 |
| PR_ES_B_1 | How often have you felt the sentiment stated in each of the sentences below?-I have someone who will listen to me when I need to talk: 3 | 0.06 | 0.1 | 0.05 | 0.14 | 0.05 | 0.17 | 0.04 | 0.21 | 0.05 | 0.13 |
| PR_ES_B_1 | How often have you felt the sentiment stated in each of the sentences below?-I have someone who will listen to me when I need to talk: 4 | 0.19 | 0.25 | 0.21 | 0.17 | 0.2 | 0.19 | 0.19 | 0.26 | 0.21 | 0.22 |
| PR_ES_B_1 | How often have you felt the sentiment stated in each of the sentences below?-I have someone who will listen to me when I need to talk: 5 | 0.73 | 0.54 | 0.72 | 0.59 | 0.73 | 0.56 | 0.73 | 0.48 | 0.71 | 0.57 |
| PR_ES_B_2 | How often have you felt the sentiment stated in each of the sentences below?-I have someone to confide in or talk to about myself or my problems: 1 | 0.01 | 0.02 | NA | NA | NA | NA | 0.01 | 0.01 | 0 | 0.01 |
| PR_ES_B_2 | How often have you felt the sentiment stated in each of the sentences below?-I have someone to confide in or talk to about myself or my problems: 2 | 0.01 | 0.09 | 0.02 | 0.05 | 0.02 | 0.06 | 0.02 | 0.06 | 0.03 | 0.04 |
| PR_ES_B_2 | How often have you felt the sentiment stated in each of the sentences below?-I have someone to confide in or talk to about myself or my problems: 3 | 0.06 | 0.11 | 0.06 | 0.14 | 0.05 | 0.14 | 0.05 | 0.17 | 0.06 | 0.12 |
| PR_ES_B_2 | How often have you felt the sentiment stated in each of the sentences below?-I have someone to confide in or talk to about myself or my problems: 4 | 0.2 | 0.22 | 0.2 | 0.2 | 0.2 | 0.21 | 0.19 | 0.27 | 0.19 | 0.27 |
| PR_ES_B_2 | How often have you felt the sentiment stated in each of the sentences below?-I have someone to confide in or talk to about myself or my problems: 5 | 0.72 | 0.56 | 0.72 | 0.59 | 0.73 | 0.56 | 0.73 | 0.48 | 0.72 | 0.55 |
| PR_ES_B_3 | How often have you felt the sentiment stated in each of the sentences below?-I have someone who makes me feel appreciated: 1 | 0.01 | 0.04 | 0.01 | 0.04 | 0.01 | 0.03 | 0.01 | 0.01 | 0.01 | 0.01 |
| PR_ES_B_3 | How often have you felt the sentiment stated in each of the sentences below?-I have someone who makes me feel appreciated: 2 | 0.03 | 0.04 | 0.02 | 0.05 | 0.03 | 0.05 | 0.02 | 0.05 | 0.03 | 0.03 |
| PR_ES_B_3 | How often have you felt the sentiment stated in each of the sentences below?-I have someone who makes me feel appreciated: 3 | 0.06 | 0.16 | 0.07 | 0.13 | 0.06 | 0.14 | 0.07 | 0.13 | 0.06 | 0.17 |
| PR_ES_B_3 | How often have you felt the sentiment stated in each of the sentences below?-I have someone who makes me feel appreciated: 4 | 0.19 | 0.18 | 0.19 | 0.19 | 0.17 | 0.2 | 0.17 | 0.29 | 0.18 | 0.24 |
| PR_ES_B_3 | How often have you felt the sentiment stated in each of the sentences below?-I have someone who makes me feel appreciated: 5 | 0.71 | 0.57 | 0.71 | 0.59 | 0.73 | 0.58 | 0.73 | 0.52 | 0.71 | 0.55 |
| PR_ES_B_4 | How often have you felt the sentiment stated in each of the sentences below?-I have someone to talk with when I have a bad day: 1 | 0 | 0.03 | 0 | 0.02 | 0 | 0.01 | NA | NA | NA | NA |
| PR_ES_B_4 | How often have you felt the sentiment stated in each of the sentences below?-I have someone to talk with when I have a bad day: 2 | 0.02 | 0.09 | 0.01 | 0.1 | 0.02 | 0.07 | 0.03 | 0.05 | 0.03 | 0.07 |
| PR_ES_B_4 | How often have you felt the sentiment stated in each of the sentences below?-I have someone to talk with when I have a bad day: 3 | 0.06 | 0.09 | 0.05 | 0.1 | 0.04 | 0.15 | 0.05 | 0.13 | 0.05 | 0.12 |
| PR_ES_B_4 | How often have you felt the sentiment stated in each of the sentences below?-I have someone to talk with when I have a bad day: 4 | 0.18 | 0.22 | 0.17 | 0.2 | 0.16 | 0.23 | 0.15 | 0.3 | 0.18 | 0.21 |
| PR_ES_B_4 | How often have you felt the sentiment stated in each of the sentences below?-I have someone to talk with when I have a bad day: 5 | 0.75 | 0.58 | 0.76 | 0.59 | 0.78 | 0.54 | 0.77 | 0.52 | 0.74 | 0.6 |
