# Supplementary material for: Development of multivariable models to predict perinatal depression before and after delivery using patient reported survey responses at weeks 4–10 of pregnancy
Source: BMC Pregnancy Childbirth. 2022 May 26;22:442. doi: 10.1186/s12884-022-04741-9 (PMC9137134; doi:10.1186/s12884-022-04741-9)
Supplement: Supplementary file 3 — Additional file 3. Comparison of machine learning methods. A word document containing the details of model tuning for the gradient boosting machine, logistic regression, and decision tree. [file 12884_2022_4741_MOESM3_ESM.docx]

Additional file 3: Comparison of machine learning methods

The comparison between gradient boosting machine (GBM), logistic regression (LR) and decision tree (DT) classifiers.

We used R version 3.6.3. We used the R package ‘caret’ version 6.0.84 for model fitting. For the gradient boosting machine we used the method "xgbTree" (from the package xgboost version 0.90.0.1) with the default hyper-parameter grid search (eta: 0.3/0.4; max_depth: 1/2/3; colsample_bytree: 0.6/0.8; subsample: 0.5/0.75/1; nrounds: 50/100/150). For logistic regression we used the method "glm" with the family = "binomial", this method has no hyper-parameters. For the decision tree we used the method "rpart" (from the package rPart version 4.1.15) with tuneLength = 20, so 20 different hyper-parameter values for model complexity were compared (cp: 0.00000000, 0.02631579, 0.05263158, 0.07894737, 0.10526316 0.13157895, 0.15789474, 0.18421053, 0.21052632, 0.23684211, 0.26315789, 0.28947368, 0.31578947, 0.34210526, 0.36842105, 0.39473684, 0.42105263, 0.44736842, 0.47368421, 0.50000000)

The optimal hyper-parameters were selected based on obtaining the highest AUC.

The results are:

|  |  | AUC (95% CI) | | | | |
| --- | --- | --- | --- | --- | --- | --- |
| Predictor Set | Model | Trimester 1 | Trimester 2 | Trimester 3 | After delivery 1 | After delivery 2 |
| GAD/EPDS/PRES | LR | 0.76 (0.71-0.81) | 0.70 (0.65-0.76) | 0.72 (0.67-0.77) | 0.71 (0.64-0.77) | 0.66 (0.59-0.72) |
| GAD/EPDS/PRES | GBM | 0.77 (0.74-0.80) | 0.69 (0.65-0.73) | 0.75 (0.71-0.79) | 0.72 (0.67-0.78) | 0.71 (0.66-0.76) |
| GAD/EPDS/PRES | DT | 0.62 (0.54-0.69) | 0.57 (0.49-0.65) | 0.63 (0.57-0.70) | 0.59 (0.50-0.67) | 0.62 (0.55-0.70) |

The decision tree classifier appears to perform poorly across time periods. The logistic regression and gradient boosting machine perform similarly in trimester 1 and 2 (when the prediction time is shorter). When the prediction time increases, gradient boosting machine appears to have higher discrimination (see trimester 3 and after delivery).
