# Supplementary material for: Development of multivariable models to predict perinatal depression before and after delivery using patient reported survey responses at weeks 4–10 of pregnancy
Source: BMC Pregnancy Childbirth. 2022 May 26;22:442. doi: 10.1186/s12884-022-04741-9 (PMC9137134; doi:10.1186/s12884-022-04741-9)
Supplement: Supplementary file 4 — Additional file 4. SHAP results. A word document with the SHAP results for each outcome. [file 12884_2022_4741_MOESM4_ESM.docx]

Additional file 4: SHAP results


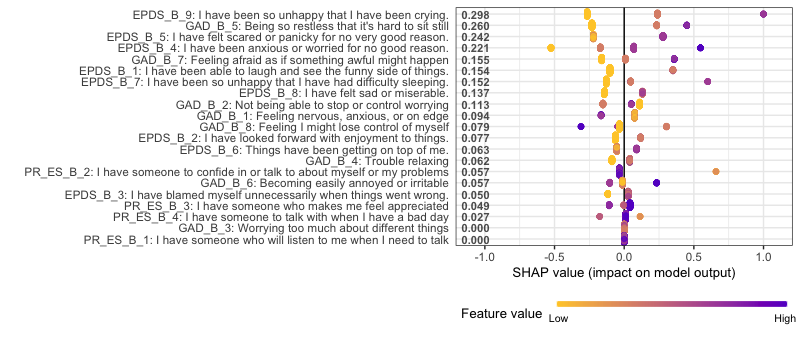


Figure 4 - Global SHAP values for baseline predictors when predicting EPDS>=12 at week 12/13

Figure 4 shows that the key predictors of a high EPDS at week 12/13 of pregnancy are answering the EPDS question at week 4-10 “I have been so unhappy that I have been crying” with yes and the GAD question “Being so restless that it’s hard to sit still” with a ‘nearly every day’ or ‘over half the days’. In general, the baseline EPDS answers seems most important in predicting EPDS at week 12/13. This suggests signs indicating depression/anxiety early in pregnancy are likely to continue for at least 3 or more weeks. The baseline PROMIS emotional support answers seems to have little impact on scoring high on EPDS at week 12/13.


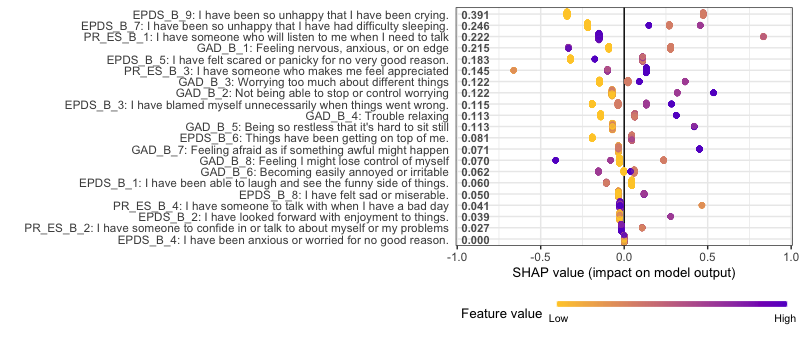


Figure 5 - Global SHAP values for baseline predictors when predicting EPDS>=12 at week 21

Figure 5 shows that answering the EPDS question at week 4-10 “I have been so unhappy that I have been crying” with yes or occasionally is highly predictive of a high EPDS score at week 21. In addition, it seems difficulty sleeping at baseline is also predictive of a high EPDS during trimester 2.


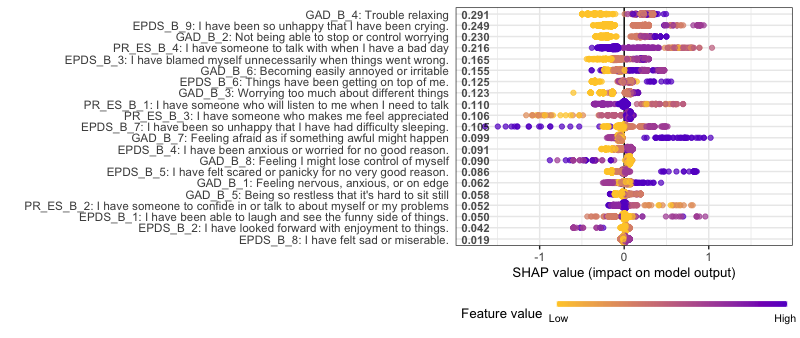


Figure 6 - Global SHAP values for baseline predictors when predicting EPDS>=12 at week 32

Figure 6 shows that the key predictors of having a high EPDS score during trimester 3 (week 32) are experiencing trouble relaxing, crying and worrying at week 4-10 of pregnancy.

In general, predictors of a high EPDS prior during pregnancy are showing signs of depression and anxiety at week 4-10 of pregnancy. This suggests that signs of peri-natal depression may show up very early into pregnancy.


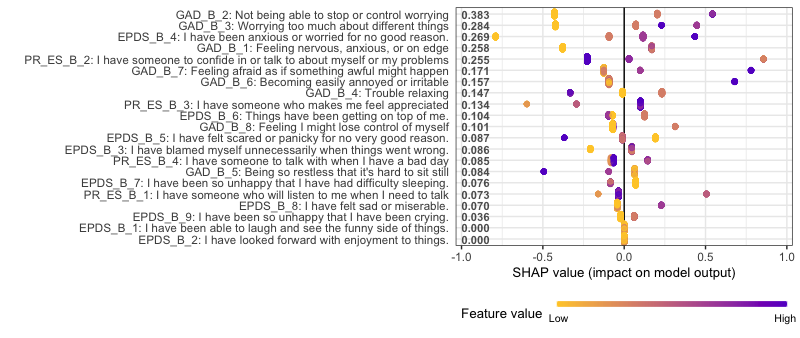


Figure 7 - Global SHAP values for baseline predictors when predicting EPDS>=12 at 4 weeks after delivery

Figure 7 shows the key baseline predictors from week 4-10 of pregnancy in predicting a high EPDS shortly after delivery (week 4 after). The main predictors are anxiety at baseline (worrying, nervousness and anxiety).


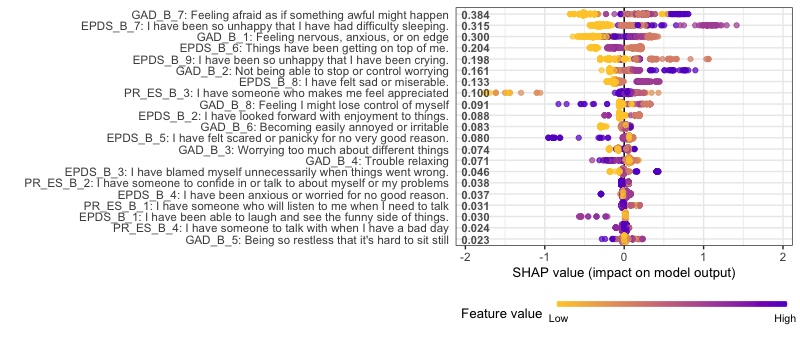


Figure 8 - Global SHAP values for baseline predictors when predicting EPDS>=12 at 12 weeks after delivery

Figure 8 shows that feeling afraid, having difficulty sleeping and feeling anxious at week 4-10 of pregnancy are predictors of a high EPDS 12 weeks after delivery.
