# Supplementary material for: Development of multivariable models to predict perinatal depression before and after delivery using patient reported survey responses at weeks 4–10 of pregnancy
Source: BMC Pregnancy Childbirth. 2022 May 26;22:442. doi: 10.1186/s12884-022-04741-9 (PMC9137134; doi:10.1186/s12884-022-04741-9)
Supplement: Supplementary file 5 — Additional file 5. Hyper-parameter search for gradient boosting machine. A word document containing the hyper-parameter grid search values for the gradient boosting machine model. [file 12884_2022_4741_MOESM5_ESM.docx]

Additional file 5: Hyper-parameter search for gradient boosting machine

The hyper-parameters for the gradient boosting machine model were learned using a grid search with the following possible values:

Nrounds (number of trees): 50, 100, 150

max_depth (max depth of each tree): 1, 2, 3

eta (learning rate): 0.3, 0.4

col sample per tree (fraction of features per tree): 0.6, 0.8

subsample (fraction of development data used by each tree): 0.25, 0.5, 0.75
